# Stable, fluorescent markers for tracking synthetic communities and assembly dynamics

**DOI:** 10.1186/s40168-024-01792-2

**Published:** 2024-05-07

**Authors:** Beatriz Jorrin, Timothy L. Haskett, Hayley E. Knights, Anna Martyn, Thomas J Underwood, Jessica Dolliver, Raphael Ledermann, Philip S. Poole

**Affiliations:** Molecular Plant Sciences Section, Department of Biology, University of Oxford, Oxford, OX1 3RB UK

**Keywords:** Synthetic community, Fluorescent labelling, Tn*7* integration, Root colonisation, Flow cytometry

## Abstract

**Background:**

After two decades of extensive microbiome research, the current forefront of scientific exploration involves moving beyond description and classification to uncovering the intricate mechanisms underlying the coalescence of microbial communities. Deciphering microbiome assembly has been technically challenging due to their vast microbial diversity but establishing a synthetic community (SynCom) serves as a key strategy in unravelling this process. Achieving absolute quantification is crucial for establishing causality in assembly dynamics. However, existing approaches are primarily designed to differentiate a specific group of microorganisms within a particular SynCom.

**Results:**

To address this issue, we have developed the differential fluorescent marking (DFM) strategy, employing three distinguishable fluorescent proteins in single and double combinations. Building on the mini-Tn*7* transposon, DFM capitalises on enhanced stability and broad applicability across diverse Proteobacteria species. The various DFM constructions are built using the pTn7-SCOUT plasmid family, enabling modular assembly, and facilitating the interchangeability of expression and antibiotic cassettes in a single reaction. DFM has no detrimental effects on fitness or community assembly dynamics, and through the application of flow cytometry, we successfully differentiated, quantified, and tracked a diverse six-member SynCom under various complex conditions like root rhizosphere showing a different colonisation assembly dynamic between pea and barley roots.

**Conclusions:**

DFM represents a powerful resource that eliminates dependence on sequencing and/or culturing, thereby opening new avenues for studying microbiome assembly.

Video Abstract

**Supplementary Information:**

The online version contains supplementary material available at 10.1186/s40168-024-01792-2.

## Background

Plant roots are colonised by a vast diversity of microorganism, with Proteobacteria and Actinobacteria amongst the most abundant groups [1–3]. These soil microorganisms are recruited in different root niches, including rhizosphere (few mm from root), rhizoplane (root surface) and endosphere (microbes between root cells) [4]. Furthermore, plants exude up to 20% of their fixed carbon into the rhizosphere thereby shaping their root microbiome, which in turn influences plant growth [5–7]. This two-way dialogue alters plant fitness, is crucial in nutrient cycling, promotes plant growth, primes plant defences and controls pathogens [3, 8–10].

The last two decades have seen an explosion in microbiome research on plants, animals and humans. Most plant studies have analysed microbiome composition by amplicon or genome sequencing under multiple conditions, including species and soil type [11–13]. More recently, use of synthetic DNA spikes enables absolute quantification of microbiome members directly in environmental samples [14]. The cutting-edge challenge is to now move beyond describing and classifying microbiomes, to understand the mechanisms of microbiome assembly. However, due to the vast diversity of microbes, this has proved to be technically challenging.

A key strategy to understand microbiome assembly is to establish a simpler representative/synthetic community (SynCom) to study and fine tune plant–microbe interactions. One of the pivotal decisions to make when designing a SynCom is the choice of size, which mainly depends on the objective of the study to perform. Vorholt et al. [15] defined that a high-complexity SynCom (more than 100 members) aims to represent the original microbiome by maintaining the diversity and thereby reducing the risk of losing keystone species and essential microbe-microbe interactions. On the other hand, in a low-complexity SynCom (less than ten members), the stochasticity is reduced, which increases experimental reproducibility and therefore it can establish a more accurate causality [15]. Most SynComs are an attempt to produce a microbial culture collection with minimal strains representative of the original phylogenetic diversity [16]. The profile, represented by the relative abundance of each strain in the assembled SynCom, is used as a phenotype under different conditions. An example is how the absence of coumarin, or the lack of cuticle biosynthesis, shifted the SynCom composition in *Arabidopsis thaliana* [17, 18]. A 185-member SynCom was used to interrogate the capacity of root growth inhibition (RGI), showing that *Variovorax* and related species within the SynCom have the capability to suppress RGI by manipulating plant hormone levels through auxin degradation [19]. SynComs can improve plant yield, as shown by the 22-member sugarcane community which displaced 54% of the natural rhizosphere microbiota and increased sugarcane fresh weight 3.4-fold compared to non-inoculated plants [20]. Whilst relative abundance quantification provides valuable insights, the power of absolute quantification reveals that specific microbial groups can maintain steady or increasing absolute abundance, even in scenarios where their relative abundances may decrease [14]. Absolute quantification emerges as a superior approach, offering a more accurate understanding of microbiome assembly dynamics and mitigating potential biases inherent in relative measurements. Nui et al. [21] measured the absolute abundance of each bacterial strain within a seven-member maize SynCom by complex culturing, including testing of 288 growth media and antibiotics combinations. The seven-membered community was stable on roots, where *Enterobacter cloacae* AA4 was a keystone species, as its absence led to collapse of the SynCom. This research highlights that one of the principal challenges in studying microbiome assembly is the identification and quantification of different bacteria during colonisation. Most SynCom studies rely on 16S RNA sequencing to describe assembly of the community, which only reveals relative microbial abundance on the roots. In contrast, differential culturing as used by Niu et al. [21] allows for experimental intervention and establishes causality in microbiome assembly, although it is labour-intensive and limited to the specific organisms for which it was developed.

Bacterial communities can be visualised and differentiate in situ by applying techniques based on the hybridisation of fluorescently labelled antisense 16S rRNA probes (FISH), which can be designed for broad groups (e.g. Actinobacteria or Betaproteobacteria), or for specific strains [2, 3, 22, 23]. FISH was applied to a seven-member SynCom in which each strain-specific probe was labelled to a particular fluorescent protein which can be distinguished by image deconvolution [24]. However, FISH has limitations such as cell loss during sample fixation and low accuracy due to an imperfect probe coverage or reduced bacterial membrane permeability [25, 26]. In small SynComs, fluorescent proteins can be expressed in bacteria; however, the limitation is the number of distinguishable ones used at the same time. Whitaker et al. [27] developed a technique with six unique fluorescent signatures by utilising two fluorescent proteins (GFP and mCherry) with different ribosome binding site (RBS)s to provide varied expression levels. When applied to a *Bacteroides* six-member SynCom colonising the guts of mice, each strain could be differentiated by linear deconvolution. Whilst this works well with strains of the same species, interspecies differentiation based on fluorescence intensity of a single fluorescent protein would require laborious tuning of expression.

The aforementioned limitations led us to develop a remarkably simple differential fluorescent marking (DFM) method using three fluorescent proteins (mTagBFP, sYFP2 and mCherry) with distinct excitation and emission spectra, allowing simultaneous detection by flow cytometry or fluorescence microscopy. Using the DFM strategy, we generate and distinguish six fluorescence patterns, i.e. three single fluorescent proteins and three combinations of two. Plasmid-based protein expression can lead to issues such as gene dosage-dependent toxicity, as well as plasmid stability and host-range. Therefore, we adapted a mini-Tn*7* delivery system [28] to generate the plasmid Tn7 suicidal low COPY for universal transfer (pTn7-SCOUT) family, enabling integration of transgenes downstream of the highly conserved chromosomal *glmS* gene in bacteria [29]. This approach is compatible with our modular and hierarchical cloning system, BEVA [30]. We tested DFM in *Rhizobium leguminosarum* bv. viciae 3841 (Rlv3841) and applied it to a six-member synthetic community (OxCom6), consisting of Alpha-, Beta- and Gammaproteobacteria. Using flow cytometry, we both differentiated and quantified the assembly of individual members of OxCom6 in nutrient-rich media and during colonisation of pea and barley roots. Our results demonstrate that DFM is an outstanding resource for tracking and distinguishing bacterial communities both in vitro, but more importantly, in diverse and complex environmental settings.

## Material and methods

### Primer and plasmids

Primer and plasmids used in this study are shown in Table S1 and Table S2, respectively. All pTn7-SCOUT plasmids are available in Addgene, see Table S3 for codes.

### Bacterial media and growth conditions

Bacterial strains used in this work are listed in Table S4. *Escherichia coli* strains were grown in LB [31] at 37 °C, supplemented with antibiotics at the following concentrations: ampicillin (Ap) 100 µg·mL^−1^, gentamicin (Gm) 10 µg·mL^−1^, kanamycin (Km) 20 µg·mL^−1^, tetracycline (Tc) 10 µg·mL^−1^ and, spectinomycin (Sp) 50 µg·mL^−1^. The remaining strains were grown in rich Tryptone Yeast (TY) media [32] supplemented with 20 mM succinate at 28 °C, unless specified otherwise. The following antibiotic concentrations were used: *Rhizobium leguminosarum* bv. viciae (Rlv3841) Gm 20 µg·mL^−1^, neomycin 40 µg·mL^−1^, Tc 5, Sp 100 µg mL^−1^; *Ochrobactrum pituitosum* AA2 and *Pseudomonas fluorescens* SBW25 Gm 20 µg·mL^−1^; *Enterobacter cloacae* AA4 Km 20 µg·mL^−1^. *Achromobacter xylosoxidans* AT1 Km 100 µg·mL^−1^ and *Azoarcus olearius* DQS-4 Sp 200 µg·mL^−1^. For the assessment of Rlv3841 labelled with DFM (Rlv3841^DFM^), mean generation time (MGT) strains were grown in UMS [33] supplemented with 10 mM glucose and 10 mM NH_4_Cl.

Plasmids were transformed into chemically competent *E*. *coli* strains DH5$\mathrm{\alpha }$, Transformax™ EC100D™ *pir*^+^ (Lucigen) and Transformax™ EC100D™ *pir-116* (Lucigen). Except for the pTn7-SCOUT plasmids which were introduced into recipient bacteria by triparental conjugation with *E*. *coli* DH5$\mathrm{\alpha }$ as plasmid donor and *E*. *coli* HB101 with the helper plasmid pRK2013 [34]. The pTn7-SCOUT plasmids were conjugated by tetraparental conjugation using *E*. *coli* Transformax™ EC100D™ *pir*^+^ as plasmid donor, *E*. *coli* S17-1 containing pTNS3 as transposase and *E*. *coli* pRK2013 as helper. Nitrofurantoin 10 µg·mL^−1^ was used to counter select against *E*. *coli* strains.

### Construction of pTn7-SCOUT plasmids

**Fig. 1:**
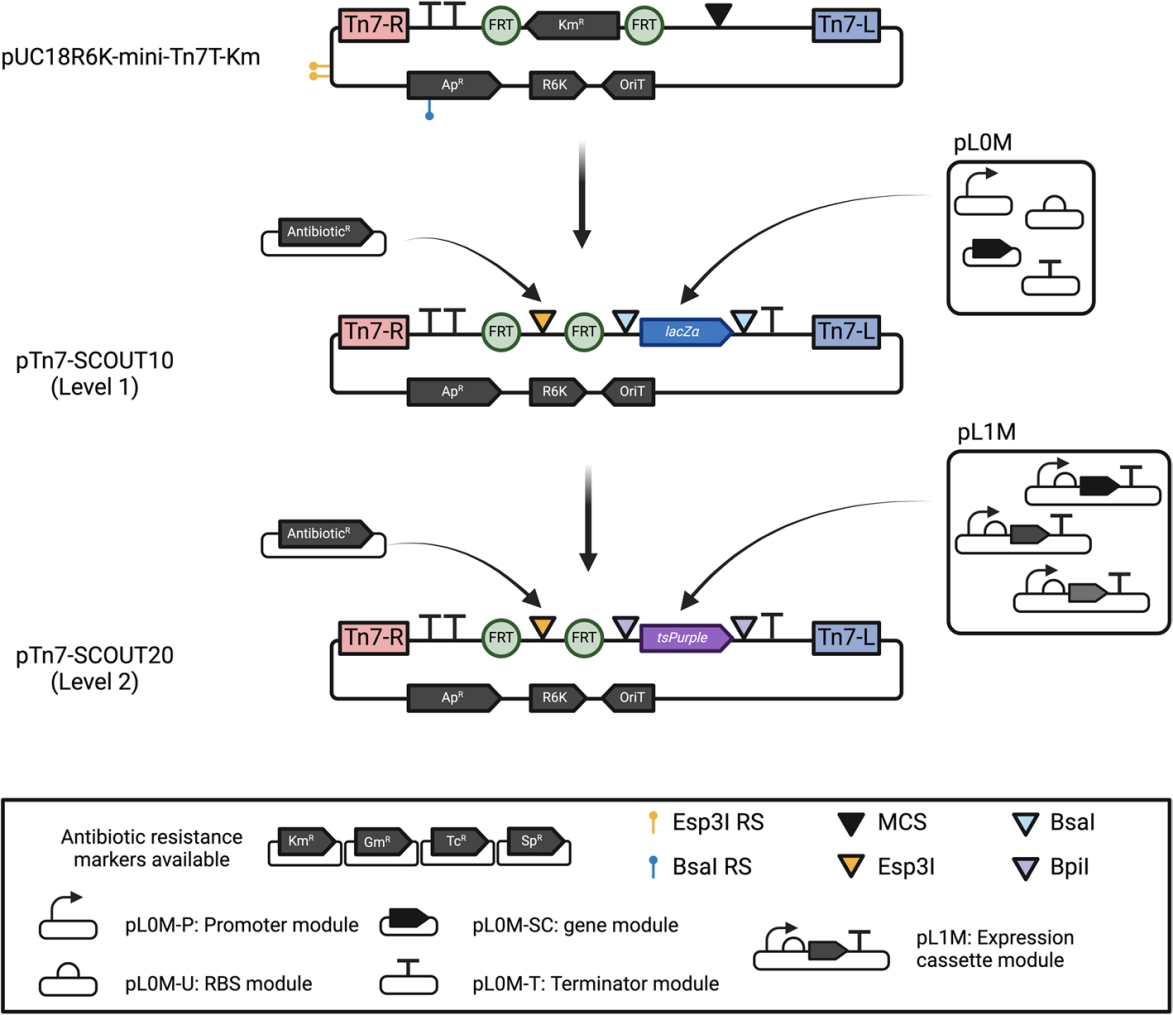
The pTn7-SCOUT plasmid family. Engineering the plasmid pUC18R6K-miniTn*7*T-Km for BEVA plasmid assembly compatibility. The plasmid was altered by removing three restriction sites (RS) and introducing two new cloning site—a designated antibiotic site and a Level 1/Level 2 site. The schematic representation includes key elements: a yellow line with a dot above indicating the Esp3I RS and blue line with a dot above indicating BsaI RS. A black inverted triangle represents the original plasmid’s multi-cloning site. Inverted blue and purple triangles depict new BsaI and BpiI sites, respectively, facilitating Golden Gate level 1 and level 2 plasmid construction. An inverted yellow triangle designates the Esp3I RS for cloning antibiotic resistance markers, located between the FRT sites (flippase recognition site, green circle). pL0M corresponds to Level 0 modules used to assemble expression cassettes in pTn7-SCOUT10. The promoter module (pL0M-P) is represented as an arrow, the ribosome binding site module (RBS, pL0M-U) as a semicircle, the gene module (pL0M-SC) as a horizontal black arrow and the terminator module (pL0M-T) represented as a “T”. pL1M corresponds to level 1 modules, representing assembled expression cassettes (promoter-RBS-gene-terminator), for constructing multiple expression cassettes in pTn7-SCOUT20. The right (Tn*7*-R) and left (Tn*7*-L) sites of the mini-Tn*7* transposon are depicted in pink and blue, respectively. The cloning marker for pTn7-SCOUT10 cloning (*lacZ*$\mathrm{\alpha }$) and for pTn7-SCOUT20 cloning (*tsPurple*) are indicated in blue and purple, respectively

The pUC18R6KT-mini-Tn*7*T-Km [28] was obtained from Addgene (catalogue no. 64969) and used as a scaffold to generate the Golden Gate level 1 master plasmid pTn7-SCOUT10. BsaI and Esp3I restrictions sites (RS) were removed, and two cloning sites added: a Golden Gate level 1 cloning site and an Esp3I cloning site to allow addition of antibiotic markers (Fig. 1). Five different fragments were generated by PCR and assembled by Golden Gate using BpiI. The first fragment was amplified using oxp3349-oxp3350 from the pUC18R6KT-mini-Tn*7*T-Km multicloning site (MCS) to the BsaI RS located in the ampicillin resistance marker (Ap^R^), changing a nucleotide in a serine codon (748A > G). The second fragment was amplified with oxp3351-oxp3352 from the BsaI RS located in Ap^R^ to two Esp3I RS located in the plasmid backbone between the Ap^R^ and R-Tn*7*. The third fragment was amplified with oxp3353-oxp3354 from the Esp3I RS in the backbone plasmid to a region between the flippase recognition site (FRT) site and 3′-end of the Km^R^. The fourth fragment was amplified with oxp3355-oxp3356 from the region between 5′-end of Km^R^ and FRT site to the mini-Tn*7* MCS. The fifth fragment was amplified with oxp2980-oxp2981 from pOGG093 plasmid [30], which amplifies the Golden Gate level 1 cloning site containing the P_lac_::*lacZ*$\mathrm{\alpha }$-T0 region. Fragments were amplified with DNA polymerase Q5 (NEB), cleaned (GeneJet PCR purification kit, Thermo Fisher), assembled by Golden Gate with BpiI as described by Geddes et al. [30], cloned in Transformax™ EC100D™ *pir-116* (Lucigen), miniprepped, and Sanger sequenced. pTn7-SCOUT10 has BsaI RS compatible with Golden Gate level 1 assembly and *lacZ*$\mathrm{\alpha }$ as cloning marker, resulting in blue/white colony colour selection when plated on media supplemented with X-gal 50 µg·mL^−1^.

To generate the Golden Gate level 2 master plasmid pTn7-SCOUT20, a new selection marker was constructed. The chromogenic gene *tsPurple* expression cassette was amplified from pOPS1522 with oxp4051-oxp4052, cloned into pTn7-SCOUT10 by Golden Gate using BsaI, transformed in Transformax™ EC100D™ *pir-116* (Lucigen), miniprepped and Sanger sequenced. pTn7-SCOUT20 has BpiI RS compatible with Golden Gate level 2 assembly and *tsPurple* as cloning marker, resulting in purple/white colony colour selection.

The antibiotic resistance cassettes within the mini-Tn*7* were cloned in pTn7-SCOUT10 and pTn7-SCOUT20 by a Golden Gate reaction using Esp3I. The pLVC-P2 modules of the gentamicin resistance marker (Gm^R^, pOGG009), tetracycline resistance marker (Tc^R^, pOGG042) and kanamycin resistance marker (Km^R^, pOGG008) were used [30]. The spectinomycin resistance marker (Sp^R^) was amplified with oxp3357-oxp3358 from pUC18T-mini-Tn*7*T-*aad9* [35] and cloned by Golden Gate reaction with Esp3I. A family of pTn7-SCOUT plasmids was generated: level 1 pTn7-SCOUT11 (Gm^R^), pTn7-SCOUT12 (Km^R^), pTn7-SCOUT13 (Tc^R^), pTn7-SCOUT14 (Sp^R^); and level 2 pTn7-SCOUT21 (Gm^R^), pTn7-SCOUT22 (Km^R^), pTn7-SCOUT23 (Tc^R^), pTn7-SCOUT24 (Sp^R^) (see Table 1, Table S2).

### Development of compatible flippase plasmids

The antibiotic marker within the mini-Tn*7* is flanked by FRT sites, allowing its excision from the chromosome by yeast recombinase flippase (Flp) following mini-Tn*7* insertion [28]. The pFLP2 plasmid [36] with Amp^R^ was obtained from Herbert P. Schweizer. The *sacB*-*flp*-*cI* genes were amplified with oxp3417-oxp3418, purified and assembly by Golden Gate with BsaI into the destination vectors pOGG024 (Gm^R^), pOGG023 (Km^R^) and pOGG277 (Tc^R^). Three new pFLP2 plasmids were generated pFlp-Km (pOPS1466; flp-cl-sacB-pL1V-Lv1-neo-pBBR1-ELT3), pFlp-Gm (pOPS1467; flp-cl-sacB-pL1V-Lv1-gent-pBBR1-ELT3) and pFlp-Tc (pOPS1468; flp-cl-sacB-pL1V-Lv1-TetAR-pBBR1-ELT3), (Table 1, Table S2).

### Assembly of Golden Gate plasmids

Assembly of plasmids was done by Golden Gate as described by Geddes et al. [30]. Esp3I was used for the assembly of level 1 cloning plasmids (pL1V-Lv1), BsaI for the assembly of the expression cassette into level 1 plasmids and BpiI for assembly of level 1 modules into level 2 plasmids. Specific details about each plasmid construction are described in Supplementary Methods.

### *att* amplification, sequencing and analysis

DNA extraction from each DFM strain was achieved by alkaline lysis (0.05 M NaOH, 0.25% SDS) [37], and used as a template to amplify by PCR the region from the 3′-end of *glmS* to Tn*7*-R. Primer PTn*7*R [28] on Tn*7*-R was used as a reverse primer and a specific forward primer was designed for each strain (see Table S1). Amplification was carried out in a 50 µL PCR reaction containing 5–10 ng of isolated DNA and 2 U of Q5 DNA polymerase (NEB). PCR products were visualised on 1% agarose gels, purified (Monarch® PCR & DNA Cleanup kit, NEB) and Sanger sequenced (Eurofins). Alignment of sequences was performed using MUSCLE [38] implemented in MEGA X software [39]. The alignment consensus was calculated in Jalview [40].

### Development and assessment of landing pad introduction into strains

To construct the *Sinorhizobium meliloti* CL150 containing the landing pad (*Sm*LP), we followed the same procedure as described by Haskett et al. [41]. Firstly, a 282 bp fragment containing the Tn7 *attB* site using oxp3192 and oxp3193 primers was PCR-amplified from Rlv3841 chromosome (Table S1). Secondly, 1 kb DNA fragments of two flanking regions of the harbour site [42] of *S*. *meliloti* CL150 were amplified using primer pairs oxp3190-oxp3191 and oxp3194-oxp3195. These three fragments were assembled by HiFi (NEB) with pK19mobSacB digested with SmaI, resulting in plasmid pOPS1246. Plasmid pOPS1246 was introduced into *S*. *meliloti* CL150, and sucrose selection [43] was used to stably integrate the Tn7 *attB* site of Rlv3841 (landing pad) into a harbour site in the chromosome by homologous recombination, resulting in *Sm*LP strain.

To test mini-Tn*7* integration specificity into the landing in *Azorhizobium caulinodans* ORS571 containing landing pad (AcLP) and *Sm*LP, two sets of primer pairs were used to PCR-amplify the 5′-end of the Rlv3841 *attB*-containing site fragment to Tn7-R (oxp2986 and oxp1390) and the Tn*7*-L to the 3′-end of the Rlv3841 *attB*-containing site fragment (PTn7L and oxp5053).

### Counterselection for Flp-containing plasmids

Rlv3841 containing a mini-Tn7-Gm-sfGFP (Rlv3841^G-Gm^) was conjugated with pOPS1468 (flp-cl-sacB-pL1V-Lv1-TetAR-pBBR1-ELT3), and colonies selected on TY containing Tc. Transconjugants were pooled and plated on TY supplemented with sucrose (12%). Fifty colonies were patched on TY media with and without Tc. Strains unable to grow on Tc were PCR-tested with primers oxp3878 and oxp3879, which bind between T0 and T1 and on FRT sequence. Two bands of 272 bp and 1240 bp were present in Rlv3841^G-Gm^, but only the 272 bp band in the Rlv3841^G^, which confirms excision of the Gm cassette.

### Microscopy images

Microscopy images were taken of cultures of Rlv3841^DFM^ strains growing on TY agar plates using a Leica M165FC. Detection of fluorescent proteins was as follows: mTagBFP with ET BFP filter (10,450,329, excitation: 405/20 nm, barrier: 460/40 nm) and exposure time 0.7 s; sYFP2 with ET YFP filter (10,447,410, excitation: 500/20 nm, barrier: 535/30 nm) and exposure time 1 s; and mCherry with ET mCherry filter (10,450,195, excitation: 560/40 nm, emission: 630/74 nm) and exposure time 0.2 s. Gain was set at 1 × , saturation at 1.0 and gamma at 1.01 for all images.

A mix containing equal amounts of cultures of Rlv3841^DFM^ and unlabelled were imaged with a Zeiss LSM 880 Airy Scan confocal microscope and analysed with ZEN Black v 3.6 software. To visualise fluorescent tags, mCherry was excited with a 561 nm wavelength laser and detected between 598 and 649 nm, sYFP2 was excited with a 488 nm wavelength laser and emission detected between 498 and 562 nm and mTag was excited with a 405 nm wavelength laser and emission detected between 440 and 490 nm. Two channels were used for the overlapping excitation and emission of sYFP2 and mTag. Channel one excited and detected mCherry and mTag, channel two excited and detected sYFP2.

### Flow cytometry

An Amnis® Cellstream® (Luminex Ltd.) flow cytometer with autosampler, equipped with 405 nm, 488 nm and 561 nm to excite TagBFP, sfGFP/sYFP2 and mCherry respectively, was used. Flow rates were set to low speed/high sensitivity (3.66 µL·min^−1^) and 5000–20,000 events defined by our gating parameters as Bacteria population were counted for each sample. Using Cellstream® Analysis 1.3.384 software, the Bacteria population was defined as the concentrated events area when plotting size (FSC) and granularity (SSC). The bacteria population was afterwards gated based on FSC (threshold > 0) and the aspect-ratio of SSC (threshold > 0.4) defining the Singlets population. Then Singlets events were gated based on their fluorescence emission, generating three colour populations: Red, Yellow and Blue for each fluorescent protein, mCherry, sYFP2 and TagBFP, respectively*.* The Red population are singlets events detected in the 561–611/31 channel above 550 FI units. The Yellow population are singlets events detected 488–528/46 channel above 500 FI units. The Blue population are singlets events detected in the 405–456/51 channel above 450 FI units (Fig. S1). Afterwards, we created six Combined populations defined as presence absence of Red, Yellow and Blue colour populations. R population (exclusively Red), Y (exclusively Yellow), B (exclusively Blue), RY (exclusively Red and Yellow), RB (exclusively Red and Blue) and YB (exclusively Yellow and Blue). For instance, an event will be assigned to the R population if belongs to the Red population whilst not belonging to either the Yellow or Blue population. This implies that only signals for mCherry detection were observed. The number of events·mL^−1^ (emL) was recorded for each Combined population in each sample and transformed into events·g root^−1^ (egr). All flow cytometer data is available at http://flowrepository.org, experiment codes are shown in Table S5.

### Growth curves to assess growth fitness

To calculate the MGT of each Rlv3841, strain labelled with DFM was grown in minimum media (UMS, [33]). A single colony of bacteria was streaked onto 10 mL UMS agar slopes supplemented with 10 mM glucose and 10 mM NH_4_Cl and incubated for 2 days. Cultures were resuspended in 4 mL of UMS supplemented with 10 mM glucose and 10 mM NH_4_Cl and washed three times. The OD_600nm_ was measured and 400 µL of 10^7^ cells·mL^−1^ were inoculated into 24-well plates (Vision Plate™, 4titude) and incubated in a plate reader (FLUOstar Omega, BMG Labtech) for 72 h, 700 rpm, 28 °C. MGT was calculated as the number of h it takes the population to double whilst in exponential growth phase [44].

### Inoculum preparation for pea root colonisation

A single colony of bacteria was streaked in 10 mL of TY supplemented with 20 mM succinate agar slopes in 30 mL universal tubes. For *E*. *cloacae* AA4, *O*. *pituitosum* AA2 and *P*. *fluorescens* SBW25 cultures were incubated overnight. *A*. *xylosoxidans* AT1 cultures were incubated for 1 day and *A*. *olearius* DQS-4 and Rlv3841 for 2 days. Once grown, cultures were resuspended in 4 mL of sterile 0.9% NaCl. OD_600 nm_ was measured and cultures were set at 10^9^ cells·mL^−1^. For competition and community experiments, cultures were mixed in equal ratios at 10^9^ cells·mL^−1^. Inocula were diluted to 10^5^ cells·mL^−1^ and 1 mL was added to each plant.

### Root colonisation experiment

Pea seeds were sterilised in ethanol 70% for 1 min, followed by 5 min in 3% NaClO. Barley seeds were sterilised in ethanol 70% for 1 min, followed by 5 min in 7% NaClO plus 0.1% Tween20 (Sigma-Aldrich). Seeds were washed with sterile distilled water. Pea seeds were pregerminated on agar-water 0.8% for 3 days at 23 °C in the dark, and after 3 days were transferred into sterilised boiling tubes containing fine vermiculite and 25 mL of root nutrient solution [45]. Sterilised barley seeds were transfer into boiling tubes containing fine vermiculite and 25 mL of root nutrient solution [45]. At 7 days after sterilisation, each seed was inoculated with a total of 10^5^ cells. At 7 days post-inoculation (dpi) (1 to 14 dpi for assembly dynamics experiment), plants were harvested by inverting and shaking the tubes. Roots were dipped in sterilised water to remove loosely attached vermiculite, separated from seed, and shoot by cutting the root below the seed, weighed, and transferred to 50-mL Falcon tubes. Then, 25 mL harvest solution (0.9% NaCl, 0.02% Silwet L-77) was added and vortexed at maximum speed for 1 min. Further, 1 mL was passed through 40 µm filters (FLOWMI™ cell strainers) and 100 µL of each sample was transferred to 96-well u-bottom plates for single cell quantification using Amnis® Cellstream® (Luminex Ltd.) flow cytometer.

### Quantification of background from plant roots

Uninoculated pea and barley plants were grown for 14 days, and samples were treated as described above. For each DFM population, emL was recorded and converted into egr. The values obtained were defined as root background and subtracted from total egr obtained from samples with bacterial inoculation (Table S6).

### Statistical analysis

Statistical analyses were performed on Prism 10 v10.02.

### Nitrogenase activity

Nitrogenase activity of *A*. *olearius* DQS-4 and *A. olearius* DQS-4 labelled with sYFP2 (AoDQS-4^Y^) on barley plants was assessed as described by Haskett et al. [41].

## Results

### Development of pTn7-SCOUT plasmids

Genomic integration of fluorescent markers is crucial for gene stability when studying bacteria in complex environments, due to the absence of plasmid-associated antibiotic selection [46]. However, fluorescent protein expression must be tuned to ensure sufficient levels of protein required for detection by microscopy and flow cytometry, whilst also avoiding toxicity due to overexpression. To overcome this challenge, we generated the pTn7-SCOUT (plasmid Tn7 Suicidal low COpy for Universal Transfer) as a family of mini-Tn*7* delivery plasmids that are compatible with BEVA modular Golden Gate cloning, and which only replicate in strains containing the *pir* genes [30, 47]. The pTn7-SCOUT plasmid family facilitates the chromosomal integration of multiple expression cassettes in a diverse group of Proteobacteria. This can be applied, as shown in this work, for tracking bacterial community through the quantification of fluorescent protein.

To develop the master pTn7-SCOUT10 (Fig. 1), we used the pUC18R6K-mini-Tn*7*T-Km developed by Choi et al. [28] as a scaffold. First, BsaI and Esp3I RS present in the pUC18R6K-mini-Tn*7*T-Km plasmid were mutated since BsaI and Esp3I sites are used for level 1 and antibiotic marker cloning, respectively. Secondly, the Km^R^ located in the mini-Tn*7* between the FRT sites was replaced with an Esp3I cloning site to allow for addition of different selection markers. Lastly, the MCS located in the mini-Tn*7* was substituted with a level 1 Golden Gate cloning site (*lacZ*$\mathrm{\alpha }$) for blue to white selection, which facilitates the assembly of one expression cassette by using BsaI. To enable the assembly of multiple expression cassettes, we generated the level 2 master plasmid pTn7-SCOUT20 by replacing the pTn7-SCOUT10 cloning site with a level 2 (*tsPurple*) for purple to white selection. Finally, we independently cloned the antibiotic markers, gentamicin (Gm^R^), kanamycin (Km^R^), tetracycline (Tc^R^) and spectinomycin (Sp^R^) by Golden Gate reaction into the Esp3I cloning site, generating the pTn7-SCOUT family (Table 1).

**Table 1:** pTn7-SCOUT plasmid family

Plasmid	Antb^R^	Description
pTn7-SCOUT10	Ap	pL0V-Lv1-pUC18R6KT-miniTn7 with Esp3I sites for Antb modules
pTn7-SCOUT20	Ap	pL0V-Lv2-pUC18R6KT-miniTn7 with Esp3I sites for Antb modules
pTn7-SCOUT11	Ap-Gm	pL1V-Lv1-pUC18R6KT-miniTn7-Gm with BsaI sites for cloning
pTn7-SCOUT12	Ap-Km	pL1V-Lv1-pUC18R6KT-miniTn7-Km with BsaI sites for cloning
pTn7-SCOUT13	Ap-Tc	pL1V-Lv1-pUC18R6KT-miniTn7-TetAR with BsaI sites for cloning
pTn7-SCOUT14	Ap-Sp	pL1V-Lv1-pUC18R6KT-miniTn7-Sp with BsaI sites for cloning
pTn7-SCOUT21	Ap-Gm	pL2V-Lv2-pUC18R6KT-miniTn7-Gm with BpiI sites for cloning
pTn7-SCOUT22	Ap-Km	pL2V-Lv2-pUC18R6KT-miniTn7-Km with BpiI sites for cloning
pTn7-SCOUT23	Ap-Tc	pL2V-Lv2-pUC18R6KT-miniTn7-TetAR with BpiI sites for cloning
pTn7-SCOUT24	Ap-Sp	pL2V-Lv2-pUC18R6KT-miniTn7-Sp with BpiI sites for cloning
pFlp-Km	Km	flp-cl-sacB-pL1V-Lv1-Km-pBBR1-ELT3
pFlp-Gm	Gm	flp-cl-sacB-pL1V-Lv1-Gm-pBBR1-ELT3
pFlp-Tc	Tc	flp-cl-sacB-pL1V-Lv1-TetAR-pBBR1-ELT3

*Antb*^*R*^ antibiotic resistance, *Ap* ampicillin, *Gm* gentamicin, *Km* kanamycin, *Tc* tetracycline, *Sp* spectinomycin

The existence of a FRT site on either side of the antibiotic expression cassette on mini-Tn*7* means that, following integration, the antibiotic marker can be removed using the Flp. To facilitate this, we also developed new antibiotic versions of the pFLP2 plasmid (*flp*, *cI*, *sacB* Ap^R^ [36]) (Table 1) to ensure compatibility with the strains used in this study. The *Rhizobium leguminosarum* bv. viciae 3841 (Rlv3841) containing the mini-Tn*7*-Gm-sfGFP (Rlv3841^G−Gm^) was conjugated with pOPS1468 (*flp-Ic-sacB*-Tc-pBBR) to excise the Gm^R^ from the integrated mini-Tn*7*. After sucrose selection, 100% of the strains were sensitive to Gm and the lack of a Gm^R^ was confirmed by PCR.

### Analysis of mini-Tn7 integration delivered by pTn7-SCOUT

**Fig. 2:**
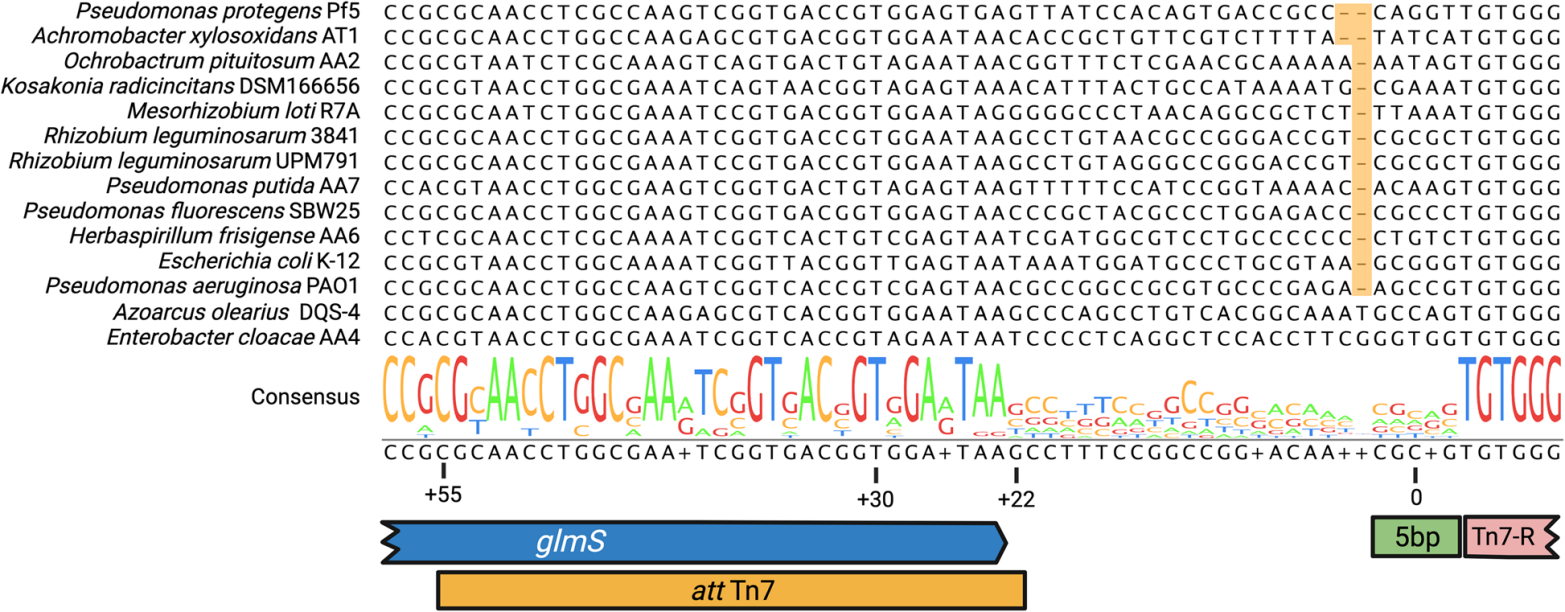
Alignment of the *att*Tn*7*-*attB* region. Nucleotide alignment of the 3′-region of the *glmS* gene—*attB* site across various tested Proteobacteria. The highlighted area in the alignment shows the differences in the distance from the stop codon of the *glmS* gene to the Tn*7* integration site (*attB*). Coordinate 0 corresponds to the central nucleotide of the 5 bp sequence that duplicates after Tn*7* integration (green rectangle). The 3′-end of the *glmS* gene is denoted by the blue arrow, whilst the *att* Tn*7* sequence is represented by the yellow rectangle. The pink rectangle signifies Tn*7*-R from the mini-Tn*7* transposon

In the model bacteria *Escherichia coli*, integration of the Tn*7* transposon occurs downstream of the *glmS* gene [48]. Different strains of Alpha-, Beta- and Gammaproteobacteria were tested for mini-Tn*7* integration delivered by pTn7-SCOUT and its integration site was assessed. The region from the 3′ end of *glmS* gene to the upstream end of the mini-Tn*7* (Tn*7*-R) was PCR amplified and sequenced (see Table S1 for primers). Nucleotide alignment of the Tn*7* integration site for these strains revealed that, as previously observed in *E*. *coli* K12 and *Pseudomonas aeruginosa* PAO1 [28, 48], Tn*7* integration occurs 25 bp from the *glmS* stop codon (Fig. 2). However, in *P*. *protegens* Pf-5 and *Achromobacter xylosoxidans* AT1, integration occurs 24 bp downstream of *glmS*, and in *Azoarcus olearius* DQS-4 and *Enterobacter cloacae* AA4 at 26 bp. Whilst 90% of the time the Tn*7* transposon integrates 25 bp downstream *glmS* in *E*. *coli* K12, it has been shown to integrate at a lower frequency, at either 24 bp or 26 bp downstream [29, 48]. Therefore, the different integration locations (*attB*) identified among the strains tested could be related to the nature of Tn*7* integration itself rather than a strain-specific effect. Upon Tn*7* integration there is a duplication of 5 bp immediately upstream to *attB* site [29]. Our results show that there is no conservation in this 5 bp sequence, suggesting that Tn*7* does not require a specific recognition sequence for integration, but rather integrates at a specific distance from the *glmS* gene (Fig. 2).

Whilst we have demonstrated that Tn*7* integration occurs 25 ± 1 bp from the *glmS* stop codon in diverse species, we found that some bacteria such *Azorhizobium caulinodans* ORS571 and *Sinorhizobium meliloti* CL150 encode a gene in this region that appear to be lethally disrupted by mini-Tn*7* insertion. We have previously overcome this issue by introducing a Tn*7* landing pad derived from the Rlv3841 Tn*7 attB* site into a neutral region of the *A*. *caulinodans* ORS571 (*Ac*LP) chromosome by double homologous recombination. This landing pad provides an alternative, non-lethal site which permits integration by Tn*7* [41]. Here, we use the same strategy to integrate the landing pad into *S*. *meliloti* CL150 chromosome at the same neutral site previously used to harbour a recombinase *attB* [42], creating strain *Sm*LP. We tested the specificity of integration into these sites for *Ac*LP and *Sm*LP with three independent conjugation experiments and were able to isolate mini-Tn*7* exconjugants of each strain harbouring the landing pad, but not for their corresponding wild-type strains, indicating the landing pads were being utilised for integration. Ten of each *Ac*LP and *Sm*LP colonies putatively harbouring mini-Tn*7* from each of the three conjugation experiments were screened by PCR using bridging across the left Tn*7 attB* site and chromosomal landing pad, confirming integration at the desired site in at least 90% for *Ac*LP (9/10, 10/10 and 9/10 colonies produced bands of the correct size) and 100% for *Sm*LP (10/10, 10/10, and 10/10 colonies produced bands of the correct size). One amplicon generated from each independent experiment was sequenced and successfully aligned to the predicted in silico sequences to further confirm this conclusion. Clearly this landing pad strategy is robust and can be applied to most strains recalcitrant to Tn*7* insertion at the native *glmS* position.

### Expression of single and dual fluorescent markers permits differentiation of up to six bacteria

The use of single fluorescent proteins to track bacteria is widely used in plant–microbe interaction studies [49, 50], but is restricted to availability of fluorophores and an ability to detect them. Our differential fluorescent marking (DFM) strategy couples use of three distinguishable fluorescent proteins, mCherry, sYFP2 and TagBFP (Fig. S2) and mini-Tn*7* stable chromosomal specific integration delivered by pTn7-SCOUT plasmids. DFM uses the aforementioned fluorescent proteins in single and double combinations to generate six unique patterns. The three single constructs are formed by cloning either, mCherry (R), sYFP2 (Y) and TagBFP (B), whilst the three doubles makers were constructed by cloning the fluorescent proteins in pairs, mCherry and sYFP2 (RY), mCherry and TagBFP (RB) and sYFP2 and TagBFP (YB).

**Fig. 3:**
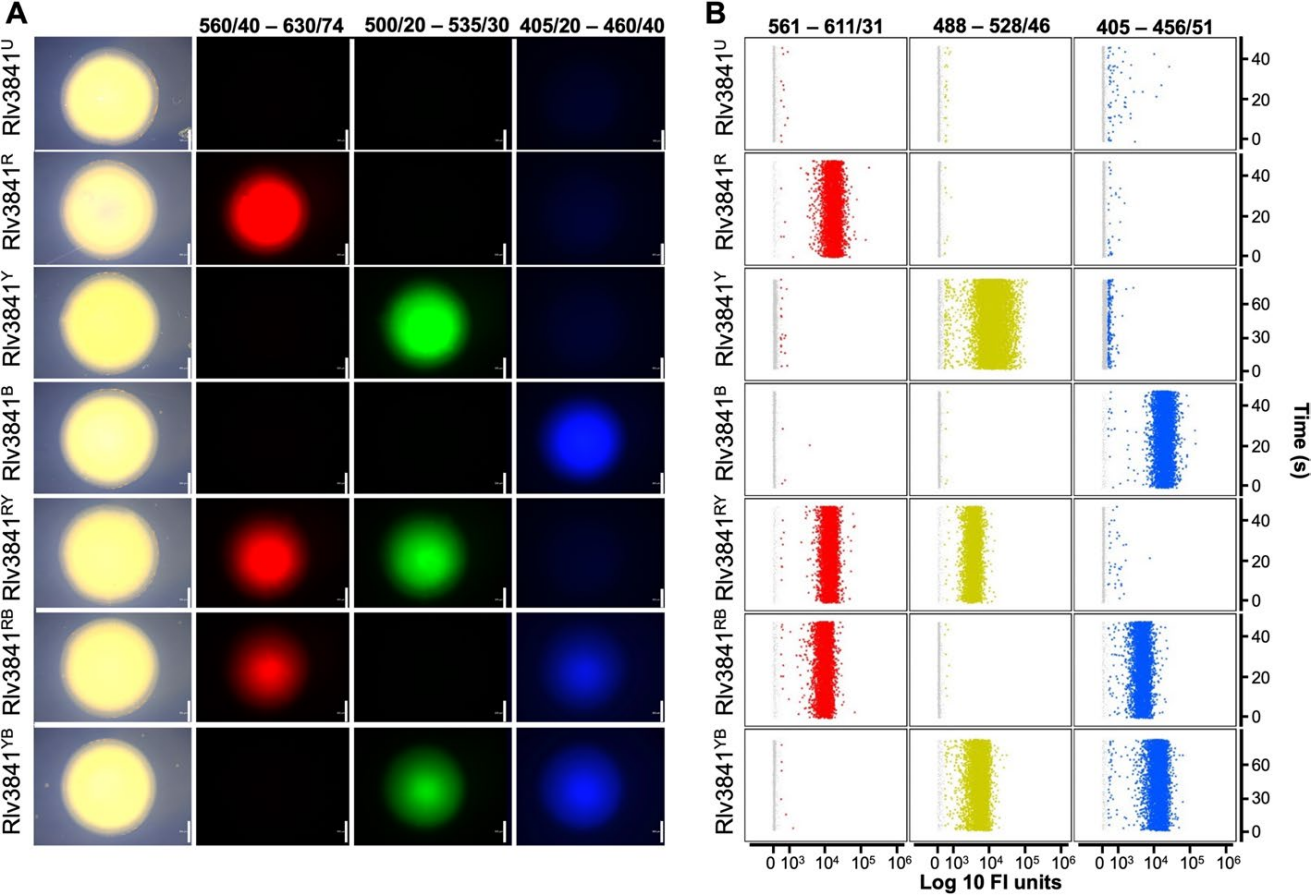
Differential Fluorescent Marking (DFM) of bacteria. Illustration of *Rhizobium leguminosarum* bv. viciae 3841 strains, unlabelled (Rlv3841^U^) and with distinct DFM combinations: Rlv3841^R^ (mCherry), Rlv3841^Y^ (sYFP2), Rlv3841^B^ (mTagBFP), Rlv3841^RY^ (mCherry and sYFP2), Rlv3841^RB^ (mCherry and mTagBFP) and Rlv3841.^YB^ (sYFP2 and mTagBFP). **A** Stereomicroscope images of Rlv3841 spots growing on rich media. The first column shows bright field images, the second 560/40–630/74 channel capturing *mCherry* expression, the third column presents the 500/20–535/30 channel capturing *sYFP2* expression and the fourth column demonstrates the 405/20–460/40 channel capturing *mTagBFP* expression. Scale bars indicate 500 µm. **B** Flow cytometry graphs, with the y-axis showing time in seconds and x-axis displaying fluorescence intensity (FI) units on a logarithmic scale. In the first column, events are plotted in 561–611/31 channel for detecting *mCherry* expression (above 550 Fi units), in the second column, events are shown in the 488–528/46 channel for detecting *sYFP2* expression (above 500 Fi units), and in the third column, events are depicted in the 405–456/51 channel for detecting *mTagBFP* expression (above 450 FI units)

To test our DFM strategy Rlv3841 was labelled with each DFM construction (Rlv3841^R^, Rlv3841^Y^, Rlv3841^B^, Rlv3841^RY^, Rlv3841^RB^ and Rlv3841^YB^) (Table 2), spotted on agar and after two days the fluorescence of each spot was detected using a fluorescent stereomicroscope, confirming the differentiation among the six DFM patterns which are not present in the unlabelled strain (Rlv3841^U^) (Fig. 3A). We expanded our investigation by combining Rlv3841^U^ and each Rlv3841^DFM^ in equal proportions. The resulting mixture was visualised using a Zeiss LSM 880 Airy Scan confocal microscope, confirming differentiation at the single-cell level among the six distinct DFM patters and unlabelled strain (Fig. S3).

**Table 2:** Description of the strains labelled with different DFM combinations

**Strain**	**Description**
Rlv3841^R^	Rlv3841 labelled with mCherry
Rlv3841^Y^	Rlv3841 labelled with sYFP2
Rlv3841^B^	Rlv3841 labelled with mTag
Rlv3841^RY^	Rlv3841 labelled with mCherry and sYFP2
Rlv3841^RB^	Rlv3841 labelled with mCherry and mTag
Rlv3841^YB^	Rlv3841 labelled with sYFP2 and mTag
OpAA2^R^	*Ochrobactrum pituitosum* AA2 labelled with mCherry
AoDQS-4^Y^	*Azoarcus olearius* DQS-4 labelled with sYFP2
AxAT1^YB^	*Achromobacter xylosoxidans* AT1 labelled with sYFP2 and mTagBFP
PfSBW25^B^	*Pseudomonas fluorescens *SBW25 labelled with mTag
EcAA4^RY^	*Enterobacter cloacae *AA4 labelled with mCherry and sYFP2

Subsequently, we ran these Rlv3841^DFM^ strains and Rlv3841^U^ independently through a flow cytometer and used Cellstream® Analysis software to distinguish the six strains based on the presence or absence of the three fluorescent proteins (Fig. 3B). First, the bacteria population was defined as the concentrated area based on size (FSC) and granularity (SSC), followed by the definition of the Singlets population based on FSC and the aspect-ration of SSC (Fig. S1A and B). Our gating strategy is followed by the delineation of three different colour population for each fluorescent marker as follows; for *mCherry* expression, the Red population as events detected 561–611/31 channel above 550 FI units; for *sYPF2* expression the Yellow population, events detected 488–528/46 channel above 500 FI units; and for *mTagBFP* expression the Blue population as the events detected in the 405–456/51 channel above 450 FI units (Fig. S1C). Afterwards, we assigned six Combined populations defined as presence or absence of the Colour populations Red, Yellow and Blue: R population (exclusively Red), Y (exclusively Yellow), B (exclusively Blue), RY (exclusively Red and Yellow), RB (exclusively Red and Blue) and YB (exclusively Yellow and Blue) (Fig. S1D). The graphs in Fig. 3B show the detection by flow cytometry of each colour population (column) for each Rlv3841^DFM^ strain (rows), which confirms the six unique DFM patters observed with the stereomicroscopy (Fig. 3A). Next, we calculated the accuracy of our flow cytometry gating strategy to assign each Rlv3841^DFM^ strain to its corresponding colour population, showing that more than 90% events were determined correctly, whereas Rlv3841^U^ showed less than 1.7% of Singlets events belonging to any of these colour population (Table 3). This 1.7% misassignment of events corresponds to events detected in the Blue colour population. The accuracy of our flow cytometry gating strategy for detecting each DFM pattern was assessed by calculating the percentage of each combined population (R, Y, B, RY, RB and YB) for each Rlv3841^DFM^ strain (Rlv3841^R^, Rlv3841^Y^, Rlv3841^B^, Rlv3841^RY^, Rlv3841^RB^ and Rlv3841^YB^). The results showed an accuracy of more than 95% in assigning the correct combined population to the corresponding DFM strain with almost complete accuracy for Rlv3841^B^ (Table 4). In this case, 99.9% of the events detected when running Rlv3841^B^ in the flow cytometer by itself were assigned as the corresponded B Combined population (Table 4). Next, we evaluated the precision of our gating strategy in discriminating each Rlv3841^DFM^ strain when present in a mixed sample, with an equal number of each strain. The number of events for each Combined population was calculated revealing that 1/6 of the total number of events were assigned to each Rlv3841^DFM^ version (Table 4).

**Table 3:** Percentage detected of each colour population for Rlv3841^U^ and each Rlv3841^DFM^ strain

DFM strain	Fluorescent population		
	**Red**	**Yellow**	**Blue**
Rlv3841^U^	0.4 ± 0.1	0.5 ± 0.2	1.7 ± 0.3
Rlv3841^R^	91.6 ± 0.4	0.2 ± 0.04	0.7 ± 0.2
Rlv3841^Y^	0.3 ± 0.02	82.9 ± 0.5	2.4 ± 0.4
Rlv3841^B^	0.1 ± 0.1	0.2 ± 0.04	92.4 ± 0.2
Rlv3841^RY^	90.8 ± 0.7	90.8 ± 0.7	1 ± 0.5
Rlv3841^RB^	86.8 ± 0.9	0.3 ± 0.1	91 ± 0.5
Rlv3841^YB^	0.2 ± 0.1	88.6 ± 0.3	90.5 ± 0.3

*n* = 3

**Table 4:** Percentage accuracy of combined population assignment to Rlv3841^DFM^ strain

DFM strain	Combined population					
	**R**	**Y**	**B**	**RY**	**RB**	**YB**
Rlv3841^R^	99.4 ± 0.2	< 0.05	0.1 ± 0.1	< 0.05	0.2 ± 0.1	0.1 ± 0.1
Rlv3841^Y^	< 0.05	97.4 ± 0.5	0.5 ± 0.1	< 0.05	< 0.05	2 ± 0.4
Rlv3841^B^	< 0.05	< 0.05	99.9 ± 0.1	< 0.05	< 0.05	< 0.05
Rlv3841^RY^	0.2 ± 0.1	< 0.05	0.7 ± 0.2	99 ± 0.1	< 0.05	0.1 ± 0.1
Rlv3841^RB^	< 0.05	< 0.05	4.5 ± 0.6	< 0.05	95.3 ± 0.6	0.1 ± 0.1
Rlv3841^YB^	< 0.05	< 0.05	2 ± 0.1	< 0.05	< 0.05	97.9 ± 0.1
Rlv3841^DFM^ (all)	15.6 ± 0.9	17.7 ± 0.5	18.3 ± 0.8	17.6 ± 0.6	16.6 ± 0.6	14.8 ± 0.8

*n* = 3

To assess if the presence of any DFM combination had a growth effect in Rlv3841, the MGT on minimum media was calculated and compared to Rlv3841^U^. No differences were observed for any of the Rlv3841^DFM^ strains, neither for each antibiotic version with a *sfGFP* expression cassette, nor for different colour combinations (Table 5). This is consistent with previous studies showing that the fluorescent protein has no effect on the fitness when integrated in single copy using mini-Tn*7* [28].

**Table 5:** Mean generation times of Rlv3841

Strain	MGT (h)	P vaule Sidak’s post hoc comparison test vs Rlv3841^U^
Rlv3841^U^	3.4 ± 0.1 (*n* = 7)	0.8849
Rlv3841^G−Gm^	3.2 ± 0.2 (*n* = 4)	0.8582
Rlv3841^G−Km^	3.2 ± 0.2 (*n* = 3)	> 0.9999
Rlv3841^G−Sp^	3.4 ± 0.2 (*n* = 5)	0.9993
Rlv3841^G−Tc^	3.5 ± 0.3 (*n* = 7)	0.727
Rlv3841^R^	3.2 ± 0.1 (*n* = 6)	0.5908
Rlv3841^Y^	3.5 ± 0.2 (*n* = 5)	0.6544
Rlv3841^B^	3.2 ± 0.2 (*n* = 4)	0.9991
Rlv3841^RY^	3.5 ± 0.1 (*n* = 6)	0.9987
Rlv3841^RB^	3.5 ± 0.2 (*n* = 6)	> 0.9999
Rlv3841^YB^	3.2 ± 0.1 (*n* = 5)	0.8849

*h* hours

To validate the use of DFM combined with flow cytometry to assess bacterial colonisation on plant roots, we inoculated Rlv3841^R^ onto pea and quantified colonisation 7 dpi by colony counts and flow cytometry. The number of Rlv3841^R^ counted with flow cytometry was 6 · 10^5^ ± 4 · 10^5^ egr and by colony count 1.1 · 10^6^ ± 8.6 · 10^5^ CFU·g root^−1^, showing no significant differences (*p* value = 0.4375, Wilcoxon test), demonstrating that flow cytometry gives comparable numbers to CFU, as shown for *Herbaspirillum* colonising rice roots [51]. Subsequently, we tested the capacity of each Rlv3841^DFM^ strain to grow on pea roots in single inoculation and in competition with Rlv3841^U^. No significant differences were observed confirming that DFM does not affect the competitive colonisation ability of the strain (Table 6). Finally, we examined the capacity to differentiate each Rlv3841^DFM^ strain when inoculated in equal amounts on pea roots. At 7 dpi, no significant differences were observed among the Rlv3841^DFM^ strains (Table 7).

**Table 6:** Colonisation of pea roots (egr) by Rlv3841^DFM^ strains when inoculated alone (single colonisation) or in competition with Rlv3841^U^ (competitive colonisation)

Strain	Single colonisation	Competitive colonisation	*P* value
Rlv3841^G−Gm^	4.7 · 10^5^ ± 3.9 · 10^5^ (*n* = 6)	4.4 · 10^5^ ± 1.6 · 10^5^ (*n* = 6)	0.84
Rlv3841^R^	2.2 · 10^6^ ± 1.6 · 10^6^ (*n* = 5)	1.7 · 10^6^ ± 1.5 · 10^6^ (*n* = 5)	0.63
Rlv3841^Y^	8.8 · 10^5^ ± 2.5 · 10^5^ (*n* = 6)	7.6 · 10^5^ ± 5.6 · 10^5^ (*n* = 5)	0.65
Rlv3841^B^	6.4 · 10^6^ ± 2.1 · 10^6^ (*n* = 5)	4 · 10^6^ ± 1.8 · 10^6^ (*n* = 4)	0.12
Rlv3841^RY^	2.7 · 10^6^ ± 1 · 10^6^ (*n* = 5)	3.2 · 10^6^ ± 2.2 · 10^6^ (*n* = 5)	0.64
Rlv3841^RB^	4.7 · 10^6^ ± 1.5 · 10^6^ (*n* = 5)	3.7 · 10^6^ ± 1.8 · 10^6^ (*n* = 4)	0.38
Rlv3841^YB^	5.6 · 10^5^ ± 3.3 · 10^5^ (*n* = 4)	6.1 · 10^5^ ± 2.9 · 10^5^ (*n* = 3)	0.84

*egr*, events ・gram root^−1^. Unpaired *t* test

**Table 7:** Colonisation of pea roots (egr) by Rlv3841^DFM^ when inoculated together in equal amounts

Strain	Events ・gram root^−1^ (egr)
Rlv3841^R^	2.1 · 10^5^ ± 1 · 10^5^
Rlv3841^Y^	1.9 · 10^5^ ± 9.5 · 10^4^
Rlv3841^B^	3 · 10^5^ ± 1.3 · 10^5^
Rlv3841^RY^	2.2 · 10^5^ ± 9.8 · 10^4^
Rlv3841^RB^	2.2 · 10^5^ ± 8.7 · 10^4^
Rlv3841^YB^	1.9 · 10^5^ ± 8.1 · 10^4^

*n* = 6. One-way-ANOVA analysis: *F* = 0.9847, *R*^*2*^ = 0.141, *p* = 0.4433

These results confirm that DFM combined with flow cytometry can be used to simultaneously differentiate and quantify up to six bacterial strains from both liquid culture and plant samples with no deleterious effects on bacterial fitness.

Since one member of OxCom6 is capable of nitrogen fixation, we tested if the presence of mini-Tn*7* affects the capacity of *A*. *olearius* DQS-4 to fix nitrogen on barley roots. The nitrogenase activity of *A*. *olearius* DQS-4 wild-type strain was 208.1 ± 44.6 nmol ethylene·plant^−1^ h^−1^, and in *A. olearius* DQS-4 integrated with mini-Tn*7* was 176.6 ± 24 nmol ethylene·plant^−1^ h^−1^. *t* Test showed no significance differences between strains (*p* value = 0.25).

### Tracking bacteria in synthetic communities using differential fluorescent markers

**Fig. 4:**
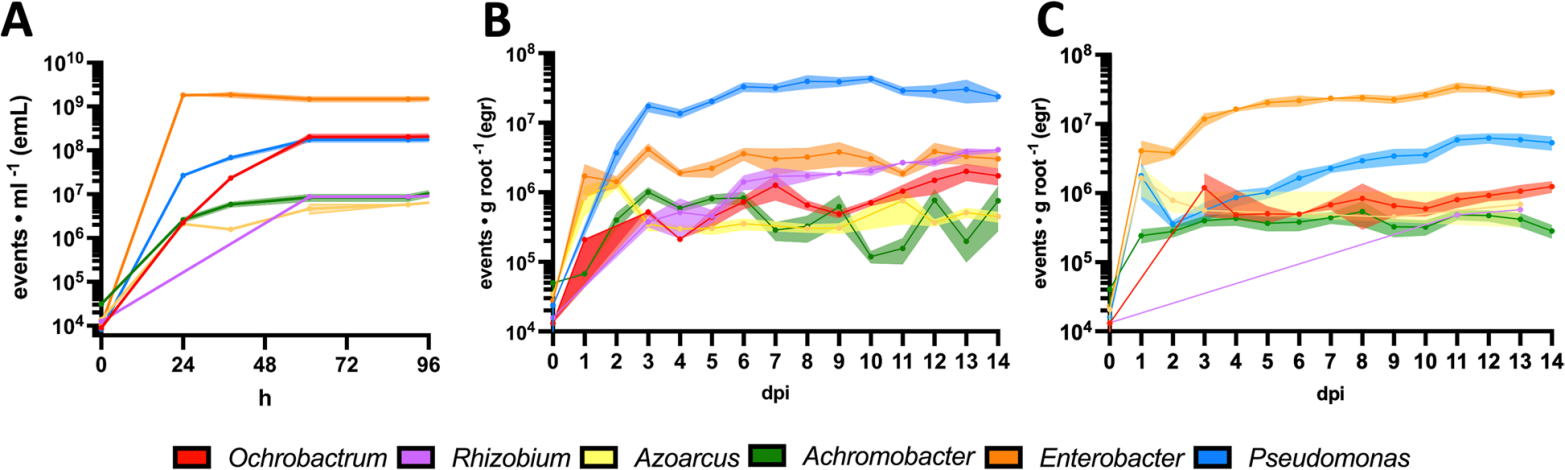
Tracking bacterial synthetic communities using DFM. Absolute quantification of each OxCom6 member: **A** growing in nutrient-rich media over 96 h, **B** colonising pea roots over 14 days and **C** colonising barley roots over 14 days. Solid dots represent the average, the shaded regions depict plus/minus the standard error of the mean (± SEM), with solid lines connecting the dots. In the graphs, members of OxCom6 are colour-coded as follows: *Ochrobactrum pituitosum* AA2^R^ (red), *Rhizobium leguminosarum* bv. viciae 3841^RB^ (purple), *Azoarcus olearius* DQS-4^Y^ (yellow), *Achromobacter xylosoxidans* AT1^YB^ (green), *Enterobacter cloacae* AA4^RY^ (orange), *Pseudomonas fluorescens* SBW25^B^ (blue). egr: events·gram root^−1^. emL: (events·mL^−^.^1^)

To test the accuracy of DFM to discriminate, track and quantify individual members in a bacterial community, a model SynCom (OxCom6) was assembled with well-characterised root-colonising strains, all of which are amenable to genetic modification. These belong to Alphaproteobacteria (*Ochrobactrum pituitosum* AA2, *R. leguminosarum* bv. viciae 3841), Betaproteobacteria (*A. xylosoxidans* AT1, *A. olearius* DQS-4) and Gammaproteobacteria (*E*. *cloacae* AA4 and *P*. *fluorescens* SBW25) [11, 21, 52–54]. Each member of the OxCom6 community was labelled with a specific DFM combination: *O*. *pituitosum* AA2 was labelled with mCherry (OpAA2^R^), *R*. *leguminosarum* bv. viciae 3841 mCherry and mTagBFP (Rlv3841^RB^), *A*. *olearius* DQS-4 sYFP2 (AoDQS-4^Y^), *A*. *xylosoxidans* AT1 sYFP2 and mTagBFP (AxAT1^YB^), *E*. *cloacae* AA4 mCherry and sYFP2 (EcAA4^RY^) and *P*. *fluorescens* SBW25 mTagBFP (PfSBW25^B^) (Table 2, and Table S4 for details on the strains used). The labelling of OxCom6 strain with each DFM pattern does not have any effect on fitness (Table S7) or competitive colonisation (Table S8). Similar to the observations for Rlv3841, when comparing CFU·mL^−1^ and events·mL^−1^ for each OxCom6 strains labelled with DFM, no differences were observed (Table S9). Subsequently, we monitored the assembly of OxCom6 in nutrient-rich media over a span of 96 h and on pea and barley roots for a duration 14 days (Fig. 4).

The results from the OxCom6 assembly in nutrient-rich media (Fig. 4A) revealed that EcAA4^RY^ exhibited a robust and sustained growth, reaching a maximum count of 1.5·10^9^ events·mL^−1^ (emL) within 24 h. In contrast, the other members of the OxCom6 reached a growth plateau at 61 h. OpAA2^R^ and PfSBW25^B^ attained peak counts of 2·10^8^ and 1.7·10^8^ emL respectively. Similarly, AxAT1^YB^ and Rlv3841^RB^ achieved comparable plateau levels, recording 8.4·10^6^ and 8.8·10^6^ emL correspondingly. Meanwhile, AoDQS-4^Y^ reached a maximum growth of 4.7·10^6^ emL. Notably, among the strains, EcAA4^RY^ demonstrated the fastest growth rate, establishing itself as the most prolific member during the OxCom6 assembly in nutrient-rich media and therefore most abundant strain when OxCom6 assembled in rich media.

Subsequently, the assembly dynamics of OxCom6 was tracked on pea roots over 14 days (Fig. 4B). At 1 dpi, EcAA4^RY^ emerged as the predominant coloniser, accounting for 10^6^ egr. However, by 2 dpi, PfSBW25^B^ displayed higher counts than EcAA4^RY^, recording figures of 3.7·10^6^ ± 3.2·10^5^ and 1.4·10^6^ ± 7.3·10^5^ egr respectively. This disparity became significant from 3 dpi with colonisation counts of 4.2·10^6^ ± 2.4·10^6^ egr for EcAA4^RY^ and 1.7·10^7^ ± 7.9·10^6^ egr for PfSBW25^B^ (paired *t* test *p* value = 0.001). Both strains achieved and sustained a plateau from 3 dpi onward, with counts *circa* 1.7–4.3·10^7^ and 1.8–4.2·10^6^ egr respectively. Starting at 10 dpi, a consistent rise was observed in the accounts of Rlv3841^RB^ and OpAA2^R^. Rlv3841^RB^ exhibited an increase from 6 to 14 dpi, rising from 1.4·10^6^ to 4.1·10^6^ egr, aligning its values with those of EcAA4^RY^. A similar pattern was evident for OpAA2^R^, which displayed growth from 4.9·10^5^ to 1.7·10^6^ egr between 9 and 14 dpi. This growth correlated positively with Rlv3841^RB^ colonisation (Pearson r = 0.91, R^2^ = 0.83, *p* value < 10^−4^). Despite early events of colonisation, the Betaproteobacteria AoDQS-4^Y^ and AxAT1^YB^ were not consistently detected within the OxCom6 assembly on pea roots. At 2 dpi, AoDQS-4^Y^ achieved a peak colonisation of 1.4·10^6^ egr. However, its counts swiftly decreased to 3.5·10^5^ egr by 3 dpi, concurring with the increase of PfSWB25^B^. This phenomenon finds support in a significant negative correlation between the two strains (Pearson r = -0.62, R^2^ = 0.38, *p* value = 0.03) indicating a potential displacement of AoDQS-4^Y^ by PfSWB25^B^. AxAT1^YB^ attained a maximum value of 1·10^6^ egr root at 3 dpi, followed by a fluctuating pattern until 14 dpi, with counts ranging between values of 10^5^·10^6^ egr.

Finally, OxCom6 assembly dynamics were tracked on barley roots over 14 days (Fig. 4C). EcAA4^RY^ emerged as the primary coloniser from 2 dpi onward, achieving a plateau of 2–3·10^7^ egr by 5 dpi. The colonisation counts of PfSBW25^B^ at 1 dpi (1.8·10^6^ ± 2.1·10^6^ egr) did not significantly differ from those observed for EcAA4^RY^ (4.1·10^6^ ± 4.4·10^6^ egr), as indicated by the paired *t* test (*p* value = 0.08). However, PfSBW25^B^ displayed a noteworthy decrease at 2 dpi (3.6·10^5^ ± 8.1·10^4^ egr), which differed significantly from the account at 1 dpi (*t* test *p* value = 0.005). Subsequently, the count of PfSBW25^B^’s rebounded to 5–6·10^6^ egr by 11 dpi when it reached plateau. Despite the early competitive events, a robust positive correlation exists between EcAA4^RY^ and PfSBW25^B^ (Pearson r = 0.81, R^2^ = 0.66, *p* value = 0.0004). OpAA2^R^ was initially detected at 3 dpi and maintained a consistent count between 5·10^5^ and 10^6^ egr up to 14 dpi. Similarly, AxAT1^YB^ exhibited a steady colonisation on barley roots, ranging 2–5·10^5^ egr. Rlv3841^RB^ was only detected at 11 and 13 dpi in one and two plants respectively, indicating that its colonisation on barley roots lacked stability in the presence of other OxCom6 members. Likewise, AoDQS-4^Y^ was detected during initial stages of colonisation (1–3 dpi) within the 5·10^5^ to 10^6^ egr range. Subsequently, it was detected at 9 and 13 dpi with roughly the same counts as before. The colonisation of AoDQS-4^Y^ at 3 dpi and 9 dpi was quantified in only one plant, and at 13 dpi in two plants. AoDQS-4^Y^ colonisation in barley roots appeared to exhibit stability during the initial colonisation events (1–3 dpi) but was subsequently outcompeted by other OxCom6 members.

## Discussion

Mini-Tn*7* is an excellent delivery system to use when working with a wide range of bacterial species in a non-selective environment since it is 100% stable for 100 generations in the absence of antibiotic selection [28, 55, 56]. Mini-Tn*7* is broad-range as demonstrated by successful delivery into multiple strains within Proteobacteria [57, 58]. Moreover, mini-Tn*7* is highly efficient and integrates in single copy into bacterial chromosomes, site- and orientation-specifically at *attB* Tn*7*, located downstream of the 3′-end of the highly conserved *glmS* gene [28]. In contrast to plasmids, mini-Tn*7* is replicated within the chromosome, therefore it does not have a fitness cost due to copy number or replication mechanism, and it is compatible with any other cloning system [59–61].

Here, we developed the pTn7-SCOUT, a new family of mini-Tn*7* plasmids compatible with the Golden Gate modular cloning system BEVA [30], which allowed us to rapidly tune the expression of the different fluorescent markers used in the DFM strategy. The pTn7-SCOUT family uses the suicidal R6K as origin of replication, which only replicates in the presence of *pir* genes supplied in *trans* [47]. Moreover, in *pir*^+^*E*. *coli* strains the R6K copy number is less than 15, which reduces the toxic effect of highly-expressed cassettes [62]. We replaced the MCS for either a level 1 or level 2 compatible Golden Gate cloning site, to allow the addition of single or multiple expression cassettes respectively. These Golden Gate cloning sites have blue/purple (*lacZ*$\mathrm{\alpha }$ / *tsPurple*) to white markers to facilitate the identification of positive transformants. The presence of a Golden Gate cloning site enables the use of a vast diversity of compatible Golden Gate modules available to construct the desired fluorescent cassette [30, 63]. Nevertheless, the pTn7-SCOUT family is not restricted to Golden Gate assembly, as the level 1 and level 2 plasmids can be digested with BsaI and BpiI respectively to become entry plasmids for classic cloning such as digestion-ligation or DNA fragment assembly methods like Gibson or HiFi (NEB). Moreover, the *lacZ*$\mathrm{\alpha }$ within the level 1 cloning site contains a polylinker for traditional cloning [30]. The pTn7-SCOUT plasmid family has an Esp3I site within the mini-Tn*7* to clone any selection marker such as antibiotic resistance genes. We successfully cloned Gm^R^, Tc^R^ and Km^R^ resistance markers using the BEVA modules [30]. However, as shown with Sp versions, any other selection marker can be cloned; by simply PCR-amplifying them with compatible overhangs, followed by cloning into pTn7-SCOUT digested with Esp3I. The level 2 Golden Gate and the antibiotic cassette cloning sites increase the modularity of the already available mini-Tn*7* delivery plasmids [28, 64]. We expanded the pTn7-SCOUT family with new antibiotic versions of Flippase-containing plasmids to enable excision of the antibiotic resistance cassette, which are compatible with the strains used in the study, since only ampicillin (Ap^R^) and Tc^R^ version were available [28, 36].

Characterization of the *attB* site has enabled us to predict the success of mini-Tn*7* integration if the host genome sequence is known. In some strains, mini-Tn*7* integration would disrupt a gene; however, we have overcome this issue by integrating a new landing pad [41], providing a new *attB* site where mini-Tn*7* is able to integrate (with an efficiency over 90% in the strains tested). This tool removes a bottleneck in mini-Tn*7* use.

The DMF tool combines single chromosomal integration with multi-fluorescence labelling to discriminate up to six different strains in a bacterial community when growing in nutrient-rich media or colonising plant roots (Fig. 4). Our flow cytometry protocol is able to discriminate with more than 95% efficiency each DFM-labelled strain (Fig. 3, Fig. S3, Table 3, Table 4, Table 7), which is as efficient as the tool developed by Whitaker et al. [27] where they combined GFP and RFP with different RBS strengths to differentiate six *Bacteroides* strains with a 6% error. The main source of misassignment detected was with the Blue Colour population (Table 3, Fig. 3B). This can be partially related to autofluorescence of aromatic amino acids, thiamine and riboflavin, detected in the 405–456/61 channel [65, 66]. However, this blue autofluorescence represents less 2% of the events in Rlv3841^U^ strain (Table 3, Fig. 3B). In addition, plant roots can show blue autofluorescence, mainly related to lignin and suberin compounds of the cell wall [67], as shown in the non-inoculated pea roots (Table S6). To overcome this issue, we quantified the background on non-inoculated pea roots for each combined population and subtracted this from the colonisation values.

High expression of fluorescent proteins can affect growth, decrease fitness, and generate toxicity due to protein aggregation and solubilisation [68, 69]. The fluorescent proteins chosen for DFM (mCherry, sYFP2 and mTagBFP) are engineered monomers with increased brightness, protein folding, extinction coefficient and maturation, which reduce deleterious effects compared to their predecessors [70–72]. Moreover, DFM is assembled in low-copy number plasmids and then integrated as a single copy into the bacterial chromosome, which reduces overall expression levels of the fluorescent proteins, and thereby any related toxicity. Furthermore, our results showed no deleterious effect of any DFM combinations during growth in liquid culture or colonisation of plants (Table 5, Table 6, Table S7, Table S8).

We successfully applied DFM to the OxCom6, a model SynCom of Proteobacteria root colonisers. Assembly of OxCom6 showed differences between nutrient-rich media, pea and barley roots (Fig. 4), indicating that the findings *in planta* can be associated with rhizosphere adaptation, as has been proven for plant microbiome [4, 73, 74].

The most marked difference was the one observed between OxCom6 assembly on pea and barley roots, where each of them have a distinct dominant strain, PfSBW25^B^ and EcAA4^RY^, respectively, and their colonisation was determined in the early stages of root occupancy (1–3 dpi) (Fig. 4 B and C). *P. fluorescens* SWB25, a well-known root coloniser isolated from sugar beet [54], is recognised to enhance plant growth through a combination of factors such as competing with other microorganisms, producing antimicrobial compounds and stimulating systemic resistance [75]. *P*. *fluorescens* SWB25 has the capability to generate furanomycin, which displays a potent inhibitory effect on the growth of *Pseudomonas*, *Bacillus*, *Erwinia* and *Dickeya* strains as observed in agar diffusion assay [76]. On the other hand, *E*. *cloacae* AA4 is part of a 7-member SynCom isolated from maize roots, and the absence of *E*. *cloacae* AA4 results in the collapse of the root colonisation by the SynCom. Whilst *E*. *cloacae* AA4 exhibits antifungal and nematocidal properties, it has not been shown to have any antibacterial activity [21, 77]. The intrinsic antibiotic capabilities of both OxCom6 Gammaproteobacteria alone do not explain the distinctive OxCom6 assembly phenotype. This suggests that there may be a rhizosphere adaptation to pea in the case of PfSBW25^B^ and to barley in the case of EcAA4^RY^, likely influenced by root exudates. The pea and barley root exudate profile have not been extensively characterised to date, but there are some studies that have provided partial descriptions of these exudates’ components. In the case of barley, a study by Calvo et al. [78] reported the presence of sugars such as sucrose, fructose and glucose at concentrations between 1 and 1.5 mg g root dry weight^−1^ at 71 days. On the other hand, the use of metabolite reporters on pea roots showed that at 4 dpi, the greater proportion of metabolites detected was sugars (xylose, fructose and myo-inositol), di-carboxylic acids (malonate and tartrate) and hesperetin; whereas, other sugars like sucrose were barely detected at this time point [79]. This suggests the different nature of pea and barley rhizosphere secretions, and therefore a different metabolic profile which the OxCom6 members can catabolise during the early stages of establishment, may be crucial in colonisation. In pea roots Rlv3841^RB^ can achieve similar levels of colonisation as EcAA4^RY^, with both reaching counts of 4.1·10^6^ egr at 14 dpi (Fig. 4B). Rlv3841 is a root symbiont of pea plants known for its unique affinity for colonising pea roots and inducing formation of nitrogen-fixing nodules [52]. Therefore, colonisation of Rlv3841^RB^ may be associated with specific niches, such as infection threads and nodules, as evident from the presence of prominent nodules formed by Rlv3841^RB^ at 13 and 14 dpi, as shown in Fig. S4. Rlv3841^RB^ root colonisation numbers on pea in OxCom6 are lower compared to those in single culture at 7dpi, 1.7·10^6^ ± 1.4·10^6^ and 4.7·10^6^ ± 1.5·10^6^ egr respectively (*t* test *p* value = 0.006) (Fig. 4B and Table 6). This suggests the potential use of OxCom6 as a controlled environment to investigate competitive colonisation of legume endosymbionts, which is critical for the competitiveness of inoculants in the field [80]. On the other hand, Rlv3841^RB^ was not detected in the barley rhizosphere (Fig. 4C), which reveals adaptation of this pea endosymbiont to its host rhizosphere [52]. Although *A. olearius* DQS-4 is capable of fixing nitrogen under free-living conditions and on barley roots, as well as promoting plant growth in rice and *Setaria viridis* [41, 81], it was not able to effectively colonise pea and barley roots in the presence of other members of OxCom6. Whilst it can colonise the root intercellular spaces of rice and *S*. *viridis*, it is not a strong competitor for pea and barley root colonisation, perhaps because it was isolated from oil-contaminated soil [53]. *O*. *pituitosum* AA2, like *E*. *cloacae* AA4, is one of the seven members of the maize SynCom and a significant contributor to that community at 14 dpi [21]. OpAA2^R^ has a strong positive correlation with the colonisation/root infection of Rlv3841^RB^ on pea roots. This could be partially facilitated by Nod factor produced by rhizobia, as legume mutants with impaired Nod factor perception have been shown to have a less abundant and altered microbiome [82, 83]. However, OpAA2^R^ showed similar root colonisation counts between pea and barley since a positive correlation was observed between both plants (Pearson r = 0.62, R^2^ = 0.39, *p* value = 0.03), which suggests a good adaptation to both plant rhizospheres, and only this strain out of the six showed any significant correlation between both plants. Therefore, the correlation with Rlv3841^RB^ on pea cannot be attributed to Rlv3841 host specificity. *A*. *xylosoxidans* AT1 was isolated from the rhizosphere of *Medicago truncatula* and it promotes growth of *A*. *thaliana*, *M*. *truncatula and Brachypodium distachyon* [11]. The fluctuating colonisation of AxAT1^YB^ on pea roots, as shown in Fig. 4B, may be influenced by stochastic availability of specific resources for bacteria in the pea rhizosphere, which can result in oscillation in bacterial growth [84, 85]. However, this is not the case on barley roots, where AxAT1^YB^ colonises in a steadier way, suggesting a better adaptation to this rhizosphere. *A*. *xylosoxidans* AT1 was isolated from *M*. *truncatula* by Tkacz et al. [11]; however, OTUs of *Achromobacter* spp. were among the most abundant in the three rhizospheres studied: *M*. *truncatula*, *A*. *thaliana* and *B*. *dystachium*. This suggests that the isolation from *M*. *truncatula* may be somewhat stochastic and does not necessarily imply that *A. xylosoxidans* AT1 is better adapted to this plant.

These results suggest that the distinct nature of the rhizosphere resources in pea and barley can result in different metabolic profiles encountered by OxCom6 members during colonisation [78, 79]. The availability of these resources in both plants would be just one aspect of the equation. Similarly, the catabolic capabilities of OxCom6 members in these rhizospheres could play a significant role in determining the assembly profile in each plant root based on their preference for catabolic sources [17, 86, 87]. However, catabolic capability alone may not be the sole determinant of this phenotype; competitive exclusion also could play a crucial role [88]. The speed at which bacteria utilise these resources could define their adaptation, and consequently, their abundance. Factors like chemotaxis and motility are pivotal in these processes [89, 90], since once a bacterium can detect a resource and effectively access and utilise it, it would gain an advantage over others, and this would lead to more rapid increase in numbers.

## Conclusion

The combination of DFM with flow cytometry allowed us to perform absolute quantification of bacterial root colonisation quickly and easily. This is crucial when assessing root colonisation dynamics, as shown in Fig. 4, since relying solely on relative abundance can lead to inaccurate comparisons between samples (Fig. S5) [14]. Whilst DFM was used here for absolute quantification of bacterial root colonisation, it can also be applied to other bacterial communities in any environment. Whilst in this study we limited the SynCom to six members to correspond to the available marker combinations, marked strains can of course be combined into larger communities. Furthermore, by varying the marked strains, large assemblies can be investigated. Techniques using DFM illustrated here provide the means for rapid assessment of microbial communities in diverse plant, animal, and environmental settings.

### Supplementary Information

**Additional file 1: Fig S1.** Flow cytometry gating strategy. Employing the CellStream® Analysis 1.3.384 software, the gating strategy was implemented to delineate the Colour and Combined populations. The initial step involved defining the Bacteria population by selecting the concentrated events area when plotting size (FSC – 456/51) against granularity (SSC – 773/56). Subsequently, the Bacteria population was gated based on FSC (threshold > 0) and the aspect-ratio of SSC (threshold > 0.4) establishing the Singlets population. Then Singlets population was further refined based on their fluorescence emission to depict the different Colour populations: Red, Yellow and Blue, corresponding to the fluorescent emission of mCherry, sYFP2 and mTagBFP, respectively. For mCherry, fluorescent emission was detected at 611/31, with a threshold above 550 FI units to define Red population. For sYFP2, emission was detected at 528/46, and the events above 500 FI units were designated as Yellow population. Emission for TagBFP was acquired at 457/51, and events exhibiting fluorescence above 450 FI units were categorised as the Blue population. Combining in one or two of the different Colour populations led to the definition of six distinct Combined populations: R (Red), Y (Yellow), B (Blue), RY (Red and Yellow), RB (Red and Blue) and YB (Yellow and Blue).

**Additional file 2: Fig S2.** Fluorescent proteins spectra. The excitation (EX) (dot) and emission (EM) (dash) spectra are shown for three fluorescent proteins: mTagBFP (blue), sYFP2 (yellow) and mCherry (red) (data sourced from fpbase.org). Vertical lines indicate the laser wavelength (nm), whilst the light bars represent the filters used in the Amnis® Cellstream® flow cytometer to detect mTagBFP (blue, 405 nm – 457/51), sYFP2 (yellow, 488 – 528/46) and mCherry (red, 561 – 528/46).

**Additional file 3: Fig S3.** Confocal microscopy images of *Rhizobium leguminosarum* 3841 unlabelled and labelled with different DFM combinations. A) bright channel. B) bright, red, yellow and blue channel. C) red, yellow and blue channel. D) red channel. E) yellow channel. F) blue channel. WT: *R. leguminosarum* 3841 (Rlv3841) not labelled. R: Rlv3841 labelled with mCherry. Y: Rlv3841 labelled with sYFP2. B: Rlv3841 labelled with mTag. RY: Rlv3841 labelled with mCherry and sYFP2. RB: Rlv3841 labelled with mCherry and mTag. YB: Rlv3841 labelled with sYFP2 and mTag.

**Additional file 4: Fig S4.** Stereomicroscope images of *Rhizobium leguminosarum* bv. viciae 3841^RB^ within nodules on pea roots inoculated with OxCom6 at 13 and 14 dpi. In the first column, bright images are shown. In the second column the 560/40—630/74 channel was utilised to observe *mCherry* expression. The third column utilises the 405/20—460/40 channel to visualise *TagBFP* expression.

**Additional file 5: Fig S5.** Absolute and relative values of community assembly of *Enterobacter cloacae* AA4. This figure represents the absolute (blue) and relative values (orange) of *E. cloacae* AA4 labelled with mCherry and sYFP2 (EcAA4^RY^) colonising pea roots (A), barley roots (B) and growing on rich media (C). egr (events•g root^−1^). emL (event•mL^−1^). Data shows that for EcAA4^RY^ that the absolute and relative values showed a different tendency on pea roots and on rich media where in both of them looks like there is a decrease when checking relative values whereas absolute values shows that the strains maintain steady.

**Additional file 6: Table S1.** Primers used in this study

**Additional file 7: Table S2.** Plasmids use in this study

**Additional file 8: Table S3.** pTn7-SCOUT plasmids developed in this study

**Additional file 9: Table S4.** Strains used in this study

**Additional file 10: Table S5.** Flow repository codes for flow cytometry data used in this study

**Additional file 11: Table S6.** Events per gram of root of non-inoculated pea and barley roots for each Combined population.

**Additional file 12: Table S7.** Mean Generation time of each OxCom6 strain unlabelled and labelled with its DFM pattern

**Additional file 13: Table S8.** Colonisation of pea roots (egr) by each OxCom6 strains when inoculated alone (single colonisation) or in competition with unlabelled strain (competitive colonisation)

**Additional file 14: Table S9. **Comparison between colony formation units and flow cytometry data for each OxCom6 strain.

**Additional file 15: Supplementary Methods.** Description of the assembly of Golden Gate plasmids used in this study.

## Data Availability

All relevant data are within the manuscript and its supporting information files. All pTn7-SCOUT plasmids are available in Addgene (See Table S3).

## Abbreviations

*Ac*LP: *Azorhizobium caulinodans* ORS571 containing the landing pad
AoDQS-4^Y^: *Azoarcus olearius* DQS-4 labelled with sYFP2
Ap: Ampicillin
Ap^R^: Ampicillin resistance marker
AxAT1^YB^: *Achromobacter xylosoxidans* AT1 labelled with sYFP2 and mTagBFP
B: Combined population with exclusively Blue colour population
DFM: Differential fluorescent marking
dpi: Days post inoculation
EcAA4^RY^: *Enterobacter cloacae* AA4 labelled with mCherry and sYFP2
egr: Events·g root^−1^
emL: Events·mL^−1^
FI: Fluorescence intensity
FISH: Fluorescently in situ hybridisation
FRT: Flippase recognition site
Gm: Gentamicin
Gm^R^: Gentamicin resistance marker
Km: Kanamycin
Km^R^: Kanamycin resistance marker
min: Minute
MGT: Mean generation time
MCS: Multicloning site
OpAA2^R^: *Ochrobactrum pituitosum* AA2 labelled with mCherry
PfSBW25^B^: *Pseudomonas fluorescens* SBW25 labelled with mTagBFP
pTn7-SCOUT: Plasmid Tn7 Suicidal low COpy for Universal Transfer
R: Combined population with exclusively Red colour population
RB: Combined population with exclusively Red and Blue colour population
RBS: Ribosome biding site
RGI: Root growth inhibition
Rlv3841: *Rhizobium leguminosarum* Bv. viciae 3841
Rlv3841^B^: Rlv3841 labelled with mTagBFP
Rlv3841^R^: Rlv3841 labelled with mCherry
Rlv3841^RB^: Rlv3841 labelled with mCherry and mTagBFP
Rlv3841^RY^: Rlv3841 labelled with mCherry and sYFP2
Rlv3841^U^: Rlv3841 not labelled
Rlv3841^Y^: Rlv3841 labelled with sYFP2
Rlv3841^YB^: Rlv3841 labelled with sYFP2 and mTagBFP
RS: Restriction site
RY: Combined population with exclusively Red and Yellow colour population
SEM: Standard error of the mean
*Sm*LP: *Sinorhizobium meliloti* CL150 containing the landing pad
Sp: Spectinomycin
Sp^R^: Spectinomycin resistance marker
SynCom: Synthetic community
Y: Combined population with exclusively Yellow colour population
YB: Combined population with exclusively Yellow and Blue colour population
Tc: Tetracycline
Tc^R^: Tetracycline resistance marker
TY: Tryptone yeast
