# Correction: Stable, fluorescent markers for tracking synthetic communities and assembly dynamics

**DOI:** 10.1186/s40168-024-01835-8

**Published:** 2024-05-17

**Authors:** Beatriz Jorrin, Timothy L. Haskett, Hayley E. Knights, Anna Martyn, Thomas J Underwood, Jessica Dolliver, Raphael Ledermann, Philip S. Poole

**Affiliations:** Molecular Plant Sciences Section, Department of Biology, University of Oxford, Oxford, OX1 3RB UK

**Correction** **:** **Microbiome 12, 81 (2024)**

**https://doi.org/10.1186/s40168-024-01792-2**

Following publication of the original article [1], the authors identified an error in the author name of Jessica Dolliver.

The incorrect author name is: Jessic Dolliver

The correct author name is: Jessica Dolliver

The author group has been updated above and the original article [1] has been corrected.
