# Supplementary material for: Stable, fluorescent markers for tracking synthetic communities and assembly dynamics
Source: Microbiome. 2024 May 7;12:81. doi: 10.1186/s40168-024-01792-2 (PMC11075435; doi:10.1186/s40168-024-01792-2)
Supplement: Supplementary file 2 — Additional file 1: Fig S1. Flow cytometry gating strategy. Employing the CellStream® Analysis 1.3.384 software, the gating strategy was implemented to delineate the Colour and Combined populations. The initial step involved defining the Bacteria population by selecting the concentrated events area when plotting size (FSC – 456/51) against granularity (SSC – 773/56). Subsequently, the Bacteria population was gated based on FSC (threshold > 0) and the aspect-ratio of SSC (threshold > 0.4) establishing the Singlets population. Then Singlets population was further refined based on their fluorescence emission to depict the different Colour populations: Red, Yellow and Blue, corresponding to the fluorescent emission of mCherry, sYFP2 and mTagBFP, respectively. For mCherry, fluorescent emission was detected at 611/31, with a threshold above 550 FI units to define Red population. For sYFP2, emission was detected at 528/46, and the events above 500 FI units were designated as Yellow population. Emission for TagBFP was acquired at 457/51, and events exhibiting fluorescence above 450 FI units were categorised as the Blue population. Combining in one or two of the different Colour populations led to the definition of six distinct Combined populations: R (Red), Y (Yellow), B (Blue), RY (Red and Yellow), RB (Red and Blue) and YB (Yellow and Blue). [file 40168_2024_1792_MOESM1_ESM.pdf]

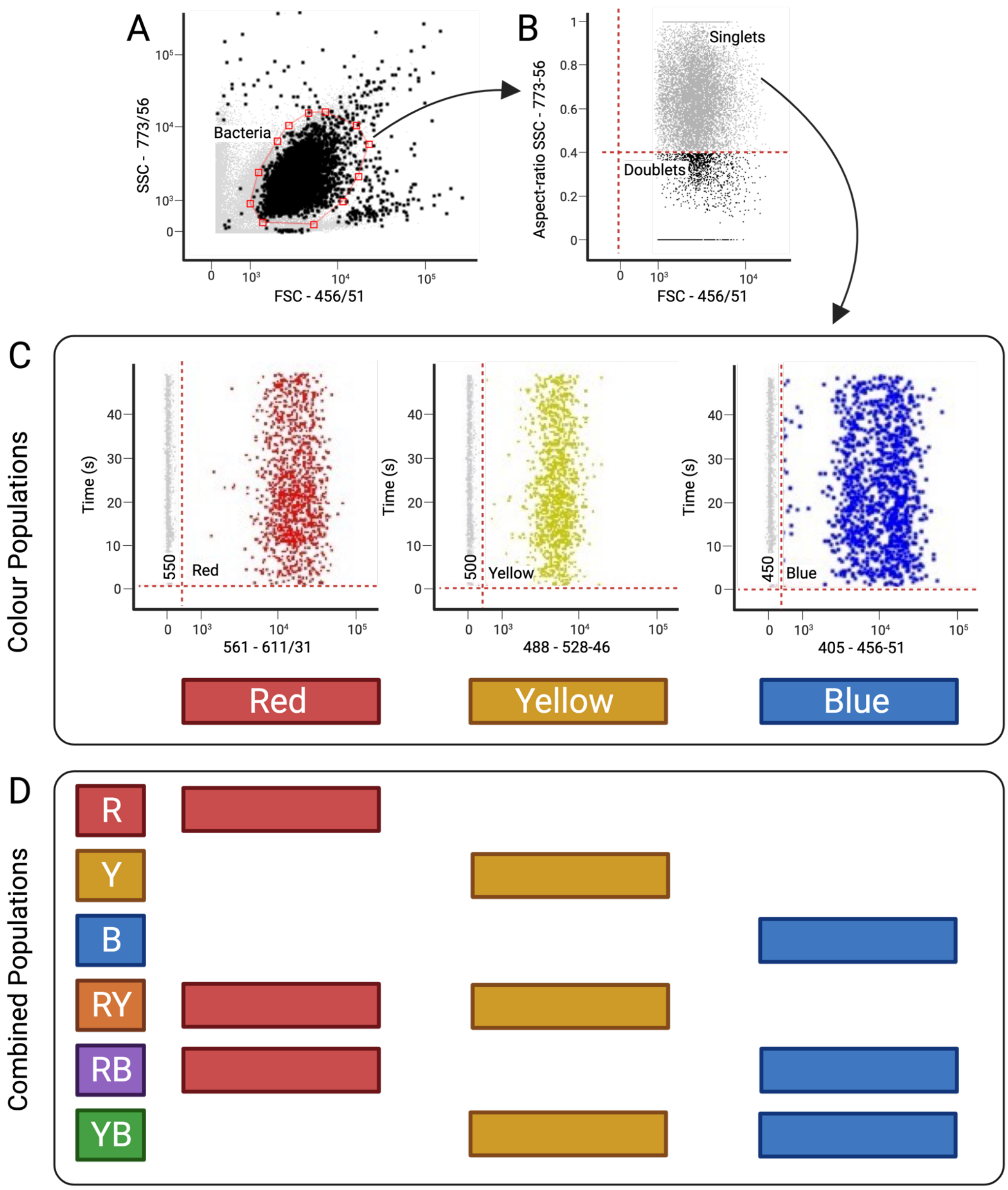

Figure S2. Flow cytometry gating strategy. Employing the CellStream® Analysis 1.3.384 software, the gating strategy was implemented to delineate the Colour and Combined populations. The initial step involved defining the Bacteria population by selecting the concentrated events area when plotting size (FSC – 456/51) against granularity (SSC – 773/56). Subsequently, the Bacteria population was gated based on FSC (threshold > 0) and the aspect-ratio of SSC (threshold >0.4) establishing the Singlets population. Then Singlets population was further refined based on their fluorescence emission to depict the different Colour populations: Red, Yellow and Blue, corresponding to the fluorescent emission of mCherry, sYFP2 and mTagBFP, respectively. For mCherry, fluorescent emission was detected at 611/31, with a threshold above 550 fluorescence intensity (FI) units to define Red population. For sYFP2, emission was detected at 528/46, and the events above 500 FI units were designated as Yellow population. Emission for TagBFP was acquired at 457/51, and events exhibiting fluorescence above 450 FI units were categorised as the Blue population. Combining in one or two of the different Colour populations led to the definition of six distinct Combined populations: R (Red), Y (Yellow), B (Blue), RY (Red and Yellow), RB (Red and Blue) and YB (Yellow and Blue).
