# Supplementary material for: Stable, fluorescent markers for tracking synthetic communities and assembly dynamics
Source: Microbiome. 2024 May 7;12:81. doi: 10.1186/s40168-024-01792-2 (PMC11075435; doi:10.1186/s40168-024-01792-2)
Supplement: Supplementary file 3 — Additional file 2: Fig S2. Fluorescent proteins spectra. The excitation (EX) (dot) and emission (EM) (dash) spectra are shown for three fluorescent proteins: mTagBFP (blue), sYFP2 (yellow) and mCherry (red) (data sourced from fpbase.org). Vertical lines indicate the laser wavelength (nm), whilst the light bars represent the filters used in the Amnis® Cellstream® flow cytometer to detect mTagBFP (blue, 405 nm – 457/51), sYFP2 (yellow, 488 – 528/46) and mCherry (red, 561 – 528/46). [file 40168_2024_1792_MOESM2_ESM.pdf]

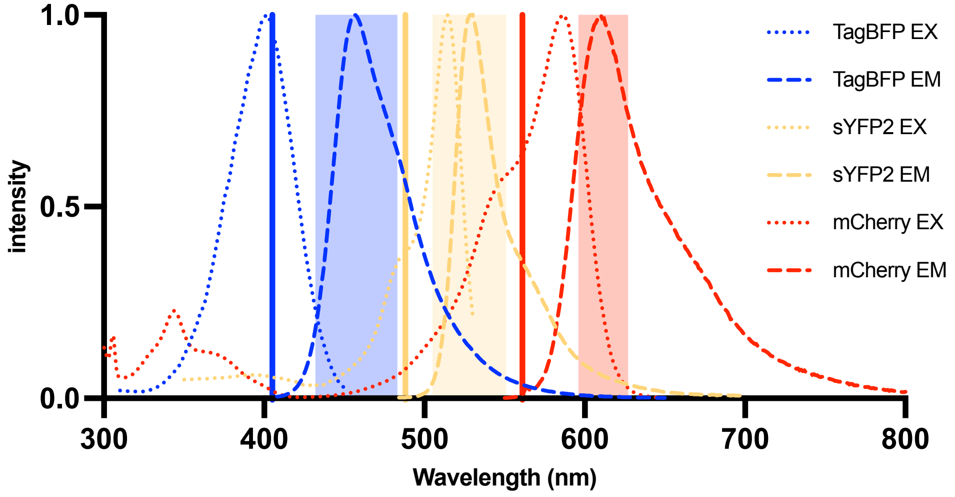

Figure S1. Fluorescent proteins spectra. The excitation (EX) (dot) and emission (EM) (dash) spectra are shown for three fluorescent proteins: mTagBFP (blue), sYFP2 (yellow) and mCherry (red) (data sourced from fpbase.org). Vertical lines indicate the laser wavelength (nm), while the light bars represent the filters used in the Amnis® Cellstream® flow cytometer to detect mTagBFP (blue, 405nm – 457/51), sYFP2 (yellow, 488 – 528/46) and mCherry (red, 561 – 528/46).
