# Supplementary material for: Stable, fluorescent markers for tracking synthetic communities and assembly dynamics
Source: Microbiome. 2024 May 7;12:81. doi: 10.1186/s40168-024-01792-2 (PMC11075435; doi:10.1186/s40168-024-01792-2)
Supplement: Supplementary file 4 — Additional file 3: Fig S3. Confocal microscopy images of Rhizobium leguminosarum 3841 unlabelled and labelled with different DFM combinations. A) bright channel. B) bright, red, yellow and blue channel. C) red, yellow and blue channel. D) red channel. E) yellow channel. F) blue channel. WT: R. leguminosarum 3841 (Rlv3841) not labelled. R: Rlv3841 labelled with mCherry. Y: Rlv3841 labelled with sYFP2. B: Rlv3841 labelled with mTag. RY: Rlv3841 labelled with mCherry and sYFP2. RB: Rlv3841 labelled with mCherry and mTag. YB: Rlv3841 labelled with sYFP2 and mTag. [file 40168_2024_1792_MOESM3_ESM.pdf]

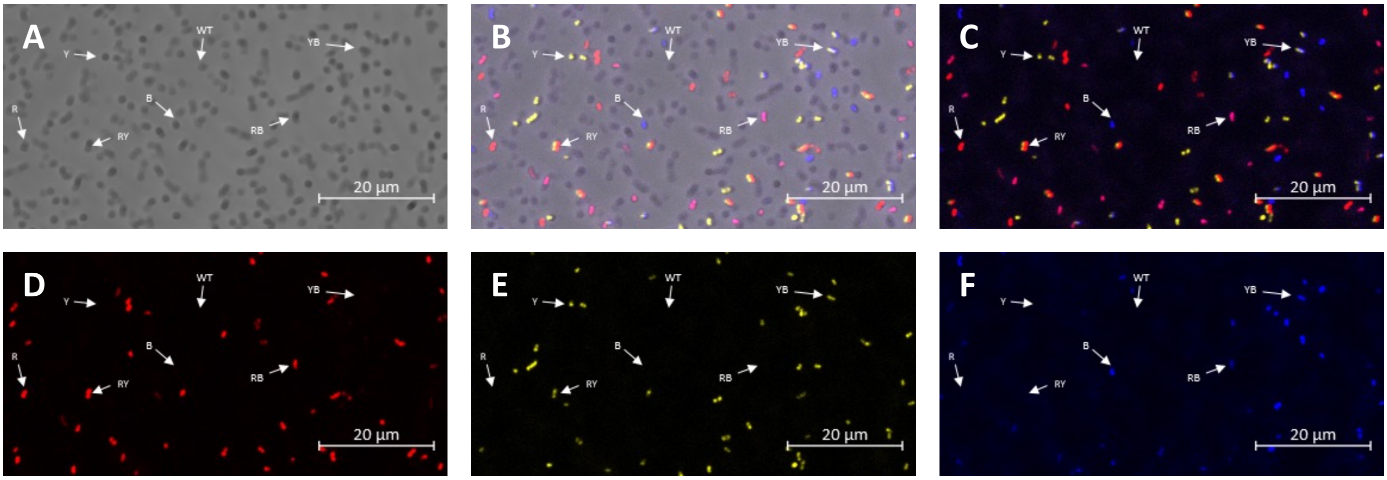


**Fig S3. Confocal microscopy images of *Rhizobium leguminosarum* 3841 unlabelled and labelled with different DFM combinations.** A) bright channel. B) bright, red, yellow and blue channel. C) red, yellow and blue channel. D) red channel. E) yellow channel. F) blue channel. WT: *R. leguminosarum* 3841 (Rlv3841) not labelled. R: Rlv3841 labelled with mCherry. Y: Rlv3841 labelled with sYFP2. B: Rlv3841 labeled with mTag. RY: Rlv3841 labelled with mCherry and sYFP2. RB: Rlv3841 labelled with mCherry and mTag. YB: Rlv3841 labelled with sYFP2 and mTag.
