# Supplementary material for: Stable, fluorescent markers for tracking synthetic communities and assembly dynamics
Source: Microbiome. 2024 May 7;12:81. doi: 10.1186/s40168-024-01792-2 (PMC11075435; doi:10.1186/s40168-024-01792-2)
Supplement: Supplementary file 5 — Additional file 4: Fig S4. Stereomicroscope images of Rhizobium leguminosarum bv. viciae 3841RB within nodules on pea roots inoculated with OxCom6 at 13 and 14 dpi. In the first column, bright images are shown. In the second column the 560/40—630/74 channel was utilised to observe mCherry expression. The third column utilises the 405/20—460/40 channel to visualise TagBFP expression. [file 40168_2024_1792_MOESM4_ESM.pdf]

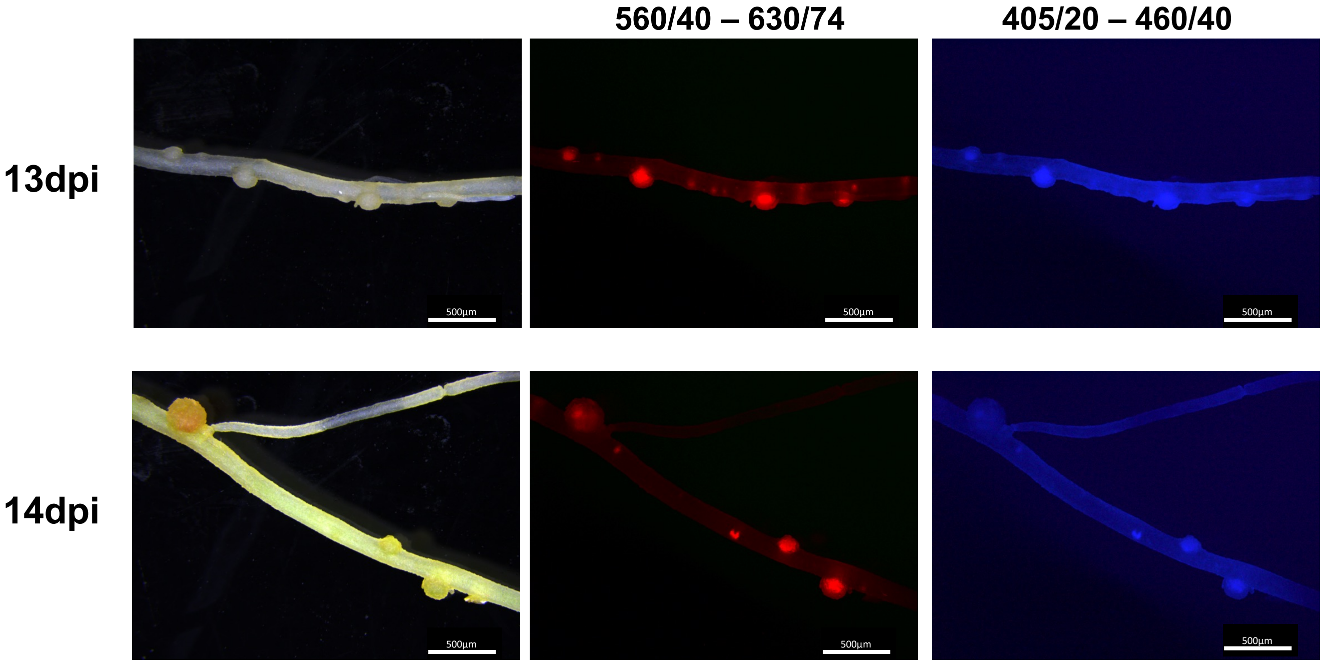

Fig S4. **Stereomicroscope images of *Rhizobium leguminosarum* bv. viciae 3841RB within nodules on pea roots inoculated with OxCom6 at 13 and 14 dpi**. In the first column, bright images are shown. In the second column the 560/40 - 630/74 channel was utilised to observe *mCherry* expression. The third column utilises the 405/20 - 460/40 channel to visualise *TagBFP* expression.
