# Supplementary material for: Stable, fluorescent markers for tracking synthetic communities and assembly dynamics
Source: Microbiome. 2024 May 7;12:81. doi: 10.1186/s40168-024-01792-2 (PMC11075435; doi:10.1186/s40168-024-01792-2)
Supplement: Supplementary file 6 — Additional file 5: Fig S5. Absolute and relative values of community assembly of Enterobacter cloacae AA4. This figure represents the absolute (blue) and relative values (orange) of E. cloacae AA4 labelled with mCherry and sYFP2 (EcAA4RY) colonising pea roots (A), barley roots (B) and growing on rich media (C). egr (events•g root−1). emL (event•mL−1). Data shows that for EcAA4RY that the absolute and relative values showed a different tendency on pea roots and on rich media where in both of them looks like there is a decrease when checking relative values whereas absolute values shows that the strains maintain steady. [file 40168_2024_1792_MOESM5_ESM.pdf]

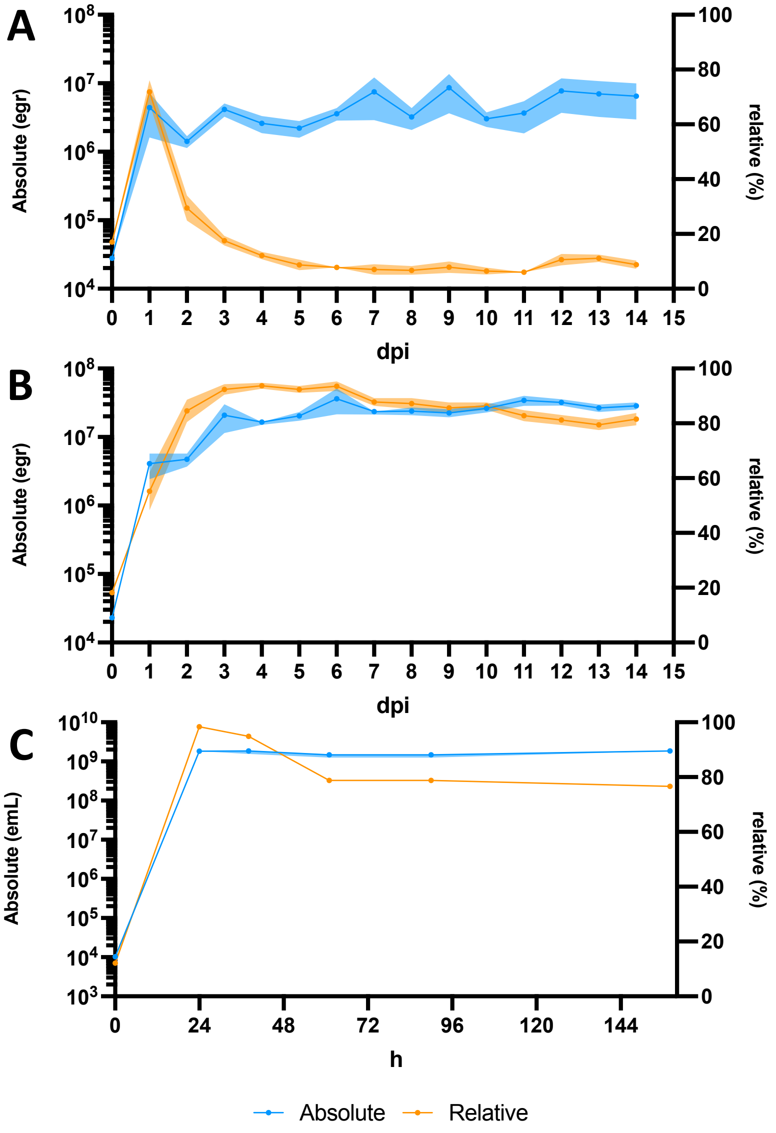

Fig S5. **Absolute and relative values of community assembly of *Enterobacter cloacae* AA4**. This figure represents the absolute (blue) and relative values (orange) of *E. cloacae* AA4 labelled with mCherry and sYFP2 ($EcAA4^{RY}$) colonising pea roots (A), barley roots (B) and growing on rich media (C). egr (events • g root^{-1}). emL (event• mL^{-1}). Data shows that for $EcAA4^{RY}$ that the absolute and relative values showed a different tendency on pea roots and on rich media where in both of them looks like there is a decrease when checking relative values whereas absolute values shows that the strains maintains steady.
