# Supplementary material for: Stable, fluorescent markers for tracking synthetic communities and assembly dynamics
Source: Microbiome. 2024 May 7;12:81. doi: 10.1186/s40168-024-01792-2 (PMC11075435; doi:10.1186/s40168-024-01792-2)
Supplement: Supplementary file 16 — Additional file 15: Supplementary Methods. Description of the assembly of Golden Gate plasmids used in this study. [file 40168_2024_1792_MOESM15_ESM.pdf]

Assembly of Golden Gate plasmids

Primer and plasmids used for the assembly of these plasmids are in Supplementary Table 1 and Supplementary Table 2, respectively.

Assembly of level 1 cloning plasmid (pL1V-Lv1)

pOGG023. Esp3I Golden Gate assembly of; [P1] pOGG004 (Golden Gate level 1 cloning site with *lacZ*); [P2] pOGG008 (kanamycin resistant); [P3] pOGG011 (pBBR1 orifin of replication and *oriT*); and [ELT3] pOGG013 (end-linker between P1 and P3).

pOGG277. Esp3I Golden Gate assembly of; [P1] pOGG004 (Golden Gate level 1 cloning site with *lacZ*); [P2] pOGG042 (tetracycline resistant); [P3] pOGG011 (pBBR1 orifin of replication and *oriT*); and [ELT3] pOGG013 (end-linker between P1 and P3).

Construction of level 1 destination plasmids (pL1V F/R RK2)

pOGG282. pL1V-F1 (colE1/RK2, pOGG021) PCR amplified with oxp3605 and oxp3606 and re-assemble by Golden Gate with Esp3I. ColE1 was removed.

pOGG284. pL1V-R2 (colE1/RK2, pOGG059) PCR amplified with oxp3605 and oxp3606 and re-assemble by Golden Gate with Esp3I. ColE1 was removed.

Construction of level 0 modules (pL0M)

pOGG179. BpiI assembly of J23100 promoter as fragment of annealed primers oxp2012 and oxp2013 with the pOGG073 pL0V-P destination vector.

pOGG297. BpiI assembly of *tsPurple* amplified from BBa_K1357008 (iGem) using oxp3913-oxp3914 primers with pOGG072 (pL0V-SC) destination vector.

Construction of pTn7-SCOUT plasmids

pTn7SCOUT11 (pOGG276). Esp3I Golden Gate assembly of pTn7-SCOUT10 (pOGG274) and gentamicin resistance module (pOGG009).

pTn7SCOUT12 (pOGG280). Esp3I Golden Gate assembly of pTn7-SCOUT10 (pOGG274) and kanamycin resistance module (pOGG008).

pTn7SCOUT13 (pOGG328). Esp3I Golden Gate assembly of pTn7-SCOUT10 (pOGG274) and tetracycline resistance module (pOGG042).

pTn7SCOUT14 (pOGG281). Esp3I Golden Gate assembly of pTn7-SCOUT10 (pOGG274) and spectinomycin resistance market (*aad9*) amplified with oxp3357-oxp3358 from pUC18T-mini-Tn*7*T-*aad9*.

pTn7SCOUT21 (pOGG299). Esp3I Golden Gate assembly of pTn7-SCOUT20 (pOGG298) and gentamicin resistance module (pOGG009).

pTn7SCOUT22 (pOGG304). Esp3I Golden Gate assembly of pTn7-SCOUT20 (pOGG298) and kanamycin resistance module (pOGG008).

pTn7SCOUT23 (pOGG342). Esp3I Golden Gate assembly of pTn7-SCOUT20 (pOGG298) and tetracycline resistance module (pOGG042).

pTn7SCOUT24 (pOGG305). Esp3I Golden Gate assembly of pTn7-SCOUT20 (pOGG298) and spectinomycin resistance market (*aad9*) amplified with oxp3357-oxp3358 from pUC18T-mini-Tn*7*T-*aad9*.

<u>Assembly of expression cassettes</u>

pOPS1522. BsaI Golden Gate assembly of; [P] pOGG120 (J23104); [U] pOGG144 (R1); [SC] pOGG297 (*tsPurple*); and [T] pOGG157 (DT16) with the pOGG026 destination vector.

pOPS1523. BsaI Golden Gate assembly of; [P] pOGG120 (J23104); [U] pOGG143 (Rstd); [SC] pOGG037 (*sfGFP*); and [T] pOGG157 (DT16) with the pTn7-SCOUT12 (pOGG280) destination vector.

pOPS1524. BsaI Golden Gate assembly of; [P] pOGG120 (J23104); [U] pOGG143 (Rstd); [SC] pOGG037 (*sfGFP*); and [T] pOGG157 (DT16) with the pTn7-SCOUT14 (pOGG281) destination vector.

pOPS1526. BsaI Golden Gate assembly of; [P] pOGG120 (J23104); [U] pOGG143 (Rstd); [SC] pOGG037 (*sfGFP*); and [T] pOGG157 (DT16) with the pTn7-SCOUT11 (pOGG276) destination vector.

pOPS1531. BsaI Golden Gate assembly of; [P] pOGG120 (J23104); [U] pOGG143 (Rstd); [SC] pOGG063 (*mCherry*); and [T] pOGG157 (DT16) with the pTn7-SCOUT11 (pOGG276) destination vector.

pOPS1568. BsaI Golden Gate assembly of; [P] pOGG120 (J23104); [U] pOGG143 (Rstd); [SC] pOGG037 (*sfGFP*); and [T] pOGG157 (DT16) with the pTn7-SCOUT13 (pOGG328) destination vector.

pOPS1606. BsaI Golden Gate assembly of; [P] pOGG120 (J23104); [U] pOGG143 (Rstd); [SC] pOGG129 (*mTagBFP*); and [T] pOGG157 (DT16) with the pTn7-SCOUT11 (pOGG276) destination vector.

pOPS1697. BsaI Golden Gate assembly of; [P] pOGG179 (J23100); [U] pOGG143 (Rstd); [SC] pOGG126 (*sYFP2*); and [T] pOGG157 (DT16) with the pTn7-SCOUT11 (pOGG276) destination vector.

pOPS1789. BsaI Golden Gate assembly of; [P] pOGG179 (J23100); [U] pOGG143 (Rstd); [SC] pOGG126 (*sYFP2*); and [T] pOGG157 (DT16) with the pTn7-SCOUT14 (pOGG281) destination vector.

pOGG300. BsaI Golden Gate assembly of; [P] pOGG120 (J23104); [U] pOGG143 (Rstd); [SC] pOGG063 (*mCherry*); and [T] pOGG157 (DT16) with the pOGG284 destination vector.

pOGG302. BsaI Golden Gate assembly of; [P] pOGG120 (J23104); [U] pOGG143 (Rstd); [SC] pOGG129 (*mTagBFP*); and [T] pOGG157 (DT16) with the pOGG282 destination vector.

pOGG303. BsaI Golden Gate assembly of; [P] pOGG120 (J23104); [U] pOGG143 (Rstd); [SC] pOGG126 (*sYFP2*); and [T] pOGG157 (DT16) with the pOGG284 destination vector.

pOGG315. BsaI Golden Gate assembly of; [P] pOGG120 (J23104); [U] pOGG143 (Rstd); [SC] pOGG126 (*sYFP2*); and [T] pOGG157 (DT16) with the pOGG282 destination vector.

<u>Assembly of multiple expression cassettes in level 2 plasmids and end-linker.</u>

pOGG273. BsaI Golden Gate assembly of ELE2 end-linker (annealed primers oxp3453 and oxp3454) with pOGG059 (pLM1-ELB2) end-linker plasmids. *lacZ* cassette was removed.

pOPS1650. BpiI Golden Gate assembly of; [F1] pOGG300 (J23104-RBstd-*mCherry*-DT16); [R2] pOGG315 (J23104-RBStd-*sYFP2*-DT16); and [ELE2] pOGG273 into pTn7-SCOUT21 (pOGG299) destination vector.

pOPS1651. BpiI Golden Gate assembly of; [F1] pOGG300 (J23104-RBstd-*mCherry*-DT16); [R2] pOGG302 (J23104-RBStd-*mTag*-DT16); and [ELE2] pOGG273 into pTn7-SCOUT21 (pOGG299) destination vector.

pOPS1652. BpiI Golden Gate assembly of; [F1] pOGG303 (J23104-RBstd-*sYFP2*-DT16); [R2] pOGG302 (J23104-RBStd-*mTag*-DT16); and [ELE2] pOGG273 into pTn7-SCOUT21 (pOGG299) destination vector.

pOPS1654. BpiI Golden Gate assembly of; [F1] pOGG300 (J23104-RBstd-*mCherry*-DT16); [R2] pOGG315 (J23104-RBStd-*sYFP2*-DT16); and [ELE2] pOGG273 into pTn7-SCOUT22 (pOGG304) destination vector.

pOPS1655. BpiI Golden Gate assembly of; [F1] pOGG303 (J23104-RBstd-*sYFP2*-DT16); [R2] pOGG302 (J23104-RBStd-*mTag*-DT16); and [ELE2] pOGG273 into pTn7-SCOUT22 (pOGG304) destination vector.
